# Assessing GPT-4o in cataract surgery decision-making: appropriateness, consistency, and clinical implications

**DOI:** 10.3389/frai.2026.1810899

**Published:** 2026-05-29

**Authors:** Yan Liu, Chaozhong Zhang, Yuanping Yuan, Kalibinur Elken, Jie Lai, Yinuo Wen, Linghao Song, Xinyue Wang, Min Zhang, Zexu Chen, Zijia Zhao, Yi Zhang, Tianhui Chen, Yongxiang Jiang

**Affiliations:** 1 Eye Institute and Department of Ophthalmology, Eye and ENT Hospital, Fudan University, Shanghai, China; 2 Key Laboratory of Myopia and Related Eye Diseases, National Health Commission, Chinese Academy of Medical Sciences, Shanghai, China; 3 Key Laboratory of Myopia and Related Eye Diseases, Chinese Academy of Medical Sciences, Shanghai, China; 4 Shanghai Key Laboratory of Visual Impairment and Restoration, Shanghai, China; 5 Department of Cataracts, Affiliated Eye Hospital of Nanchang University, Nanchang, China; 6 Central Hospital of Xinyu Iron and Steel Group, Xinyu, China; 7 Department of Cataracts, Second People's Hospital of Kashgar, Kashgar, China; 8 Eye Institute and Department of Ophthalmology, The First Affiliated Hospital of Nanchang University, Nanchang, China; 9 Eye Institute and Department of Ophthalmology, The First Affiliated Hospital of Zhejiang Chinese Medical University, Hangzhou, China

**Keywords:** appropriateness, cataract, cataract surgeon, GPT-4o, intraocular lens, large language models (LLMs)

## Abstract

**Purpose:**

To evaluate the application of large language models (LLMs) in clinical decision-making for cataract surgery using real-world patient data.

**Methods:**

This retrospective clinical observational study included 74 cataract patients (146 eyes) who underwent phacoemulsification and intraocular lens (IOL) implantation. Preoperative datasets, including clinical findings from ophthalmic imaging, biometric parameters, systemic histories, and lifestyle questionnaires, were processed via GPT-4o. The model was prompted to simulate an experienced cataract surgeon. GPT-4o's IOL recommendations were evaluated for appropriateness with an adjudicated expert consensus and compared against selections made by high- and low-volume cataract surgeons. Appropriateness rates, agreement (Kappa value), and consistency (intraclass correlation coefficient, ICC) were analyzed.

**Results:**

The overall appropriateness rate of GPT-4o for IOL selection was significantly lower than that of human surgeons (37.84% vs. 72.14%, *P* = 0.001). Subgroup analysis revealed that appropriateness varied by IOL category (*P* < 0.001), with the highest alignment observed for monofocal IOLs (90.0%) and the lowest for trifocal toric IOLs (9.09%). The presence of ocular or systemic comorbidities was associated with a slight reduction in appropriateness. While agreement analysis indicated poor inter-rater agreement (Kappa < 0.4), GPT-4o demonstrated a slightly higher ICC (0.378) than human surgeons (0.359). However, repeated testing showed significant instability in the model's responses (*P* = 0.026).

**Conclusion:**

GPT-4o demonstrated moderate appropriateness for routine monofocal IOL selection but showed limited appropriateness and consistency in complex, customized scenarios. These findings suggest GPT-4o could assist routine decision-making in cataract surgery, while highlighting the need for further domain-specific fine-tuning and multimodal optimization for advanced cases.

## 1 Introduction

Modern cataract surgery aims not only to restore visual acuity but also to improve patients' quality of life and achieve spectacle independence through precise intraocular lens (IOL) selection. Numerous IOL types—monofocal, bifocal, trifocal, toric, and extended depth of focus (EDOF)—present distinct advantages and challenges, making optimal selection complex and highly individualized (Coassin et al., 2020). Successful outcomes depend on both objective parameters, such as ocular biometry and comorbidities, as well as subjective factors including visual goals, lifestyle, and expectations (Lundstrom et al., 2018).

Despite standardized surgical guidelines, IOL selection remains largely dependent on a surgeon's experience and intuition, introducing variability and inconsistency (Kohnen, 2019). Thus, there is growing interest in developing tools to support evidence-based, personalized decision-making (Logothetis and Feder, 2019).

Artificial intelligence (AI), particularly large language models (LLMs) such as GPT-4o (OpenAI, San Francisco, CA), has demonstrated remarkable capability in integrating multimodal information for clinical reasoning (Bernstein et al., 2023; Betzler et al., 2023; Lyons et al., 2024). These models can synthesize text, numerical data, and imaging inputs, achieving near-human performance in specific diagnostic tasks (Antaki et al., 2023; Maywood et al., 2024). However, their application in real-world clinical decision-making—especially in individualized surgical planning—remains underexplored (Chen et al., 2025; Clusmann et al., 2023; Lim et al., 2023; Liu et al., 2023).

This study evaluates GPT-4o's ability to select appropriate IOL types compared with experienced cataract surgeons, examining appropriateness, consistency, and performance across patient subgroups. The results provide insights into the potential and limitations of LLMs in supporting ophthalmic clinical practice.

## 2 Methods

### 2.1 Study design and participants

**Figure 1:**
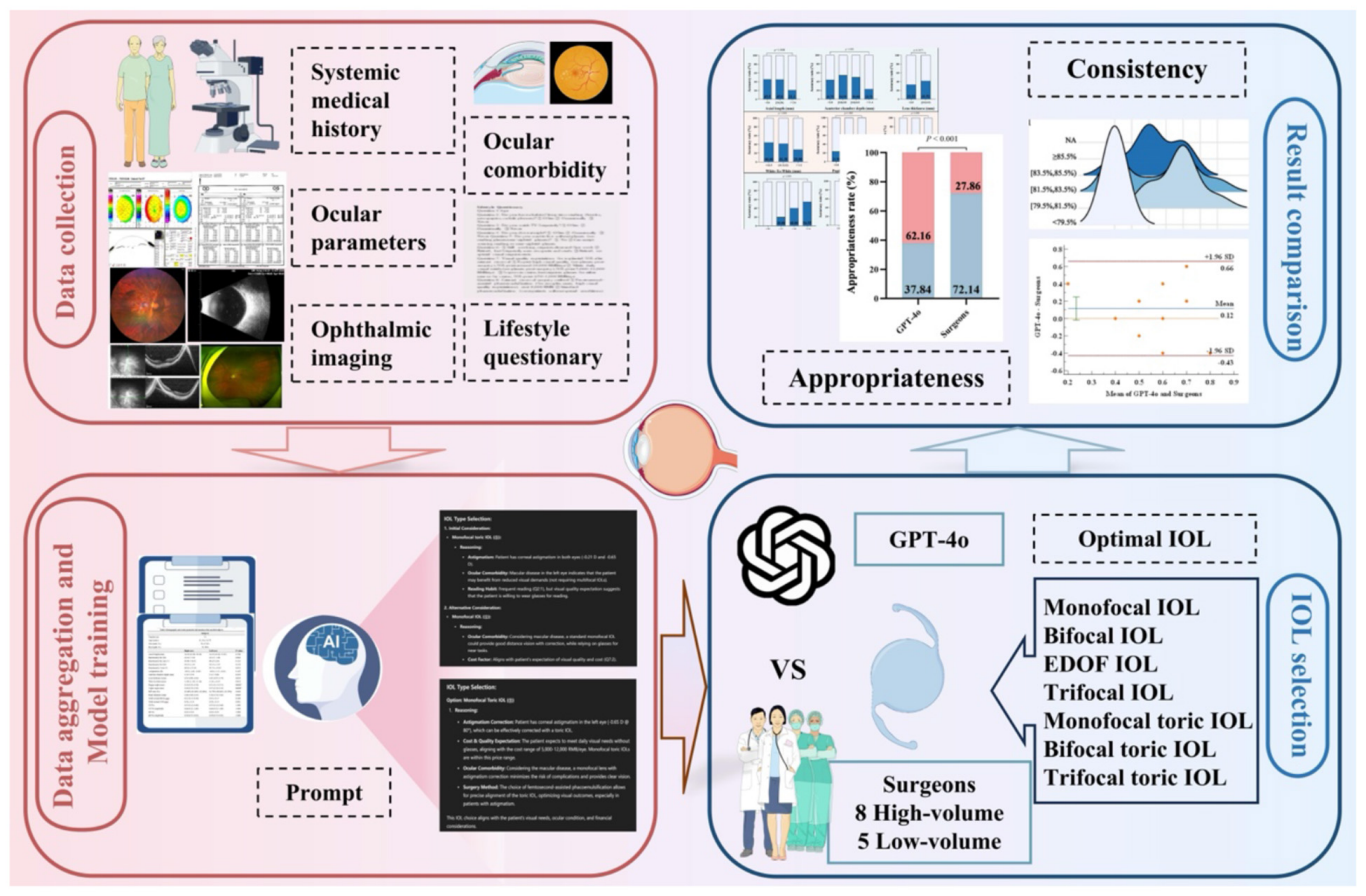
Flowchart of the study. Step 1 involves data collection. Step 2 focused on preprocessing case data into standardized prompts for GPT-4o evaluation. Step 3 consists of selecting the optimal IOL for patients by GPT-4o and different groups of surgeons. Step 4 compares the appropriateness and consistency of selections made by GPT-4o and the surgeons. EDOF, extend depth of focus; IOL, intraocular lens.

This retrospective study adhered to the Declaration of Helsinki and received approval from the Institutional Review Board of the Eye and ENT Hospital of Fudan University (Approval No. 2026-YS-208). All model queries were conducted between May 2025 and August 2025. Written informed consent was obtained from all participants or their guardians in subjects under 18 years old. Seventy-four patients (146 eyes) who underwent phacoemulsification and IOL implantation between April and June 2024 were enrolled. All surgeries were performed by Dr. Yongxiang Jiang. The study workflow is illustrated in Figure 1.

### 2.2 Data collection

Demographic characteristics (age, sex), systemic and ocular comorbidities, and prior ocular surgeries were recorded. Preoperative ocular biometry parameters such as axial length (AL), flattest and steepest keratometry (K1 and K2), anterior chamber depth (ACD), lens thickness (LT), and white-to-white (WTW) were recorded. Ocular biometrics were obtained using an IOLMaster 700 (Carl Zeiss Meditec AG, Jena, Germany) and a rotating Scheimpflug camera (Pentacam, Oculus Optikgeräte GmbH, Wetzlar, Germany). B-scan ultrasound, fundus photography (CLARUS 500, Carl Zeiss Meditec AG, Jena, Germany), and optical coherence tomography (Spectralis OCT, Heidelberg Engineering, Heidelberg, Germany, and Cirrus OCT, Carl Zeiss Meditec, Dublin, CA, United States) were also collected.

Patients completed a modified version of Dell's Cataract and Refractive Lens Exchange Questionnaire, assessing reading habits, driving needs, spectacle independence goals, and lifestyle. IOL choices were categorized into seven types: monofocal, bifocal, trifocal, EDOF, monofocal toric, bifocal toric, and trifocal toric IOLs. IOL selection is not unique, and multiple clinically acceptable options may exist for a given case. The detailed content of the questionnaire can be found in the Supplementary material 1.

All comparisons in this study were based on consistency with a predefined reference standard. The reference standard was established based on current international clinical guidelines (e.g., ESCRS/ASCRS consensus), clinical experience of senior cataract specialists, and objective ocular biometry data combined with subjective patient visual requirements. The detailed decision criteria for IOL selection are provided in Supplementary material 2. For each case, one or more IOL categories could be considered acceptable; consistency was defined as whether the selected IOL fell within this predefined acceptable range.

### 2.3 GPT-4o prompting procedure

GPT-4o (version 2024) was instructed to act as an experienced cataract surgeon. For each patient, investigators entered a standardized description summarizing all clinical data and imaging. To avoid memory bias, a new GPT-4o session was initiated for each case, with prior conversation history cleared. The chatbot selected the most suitable IOL type for each patient. Each case was repeated five times to evaluate intra-model consistency. The raw outputs from GPT-4o are provided in Supplementary material 3.

### 2.4 Statistical analysis

Statistical analyses were performed using SPSS (version 20.0; IBM Corp., NY, USA) and GraphPad Prism v9.0 (GraphPad Software, CA, USA). Continuous variables were assessed for normality; normally distributed data are expressed as mean ± SD, non-normal data as median (IQR).

The primary outcome, appropriateness of IOL selection, was defined as the alignment between GPT-4o's recommendations and the adjudicated expert consensus. Inter-rater agreement was assessed using Cohen's Kappa and ICC. Kappa values were interpreted according to the Landis and Koch criteria, whereas ICC values were interpreted using commonly accepted reliability thresholds. Group comparisons employed *t*-tests, Mann–Whitney *U*, χ^2^, or ANOVA as appropriate, with two-tailed *P* < 0.05 considered significant.

## 3 Results

### 3.1 Patient demographics

A total of 74 patients (146 eyes) were included. No statistically significant differences were found between the right and left eyes in ocular biometric parameters, except for chord μ (*P* = 0.026) and chord α (*P* = 0.018; Table 1).

Table 1Demographic and ocular parameter information of the enrolled subjects.

**Table:** 

Variable	Subjects
Number (*n*, 100%)	74 (100%)
Age (years)	61.24 ± 18.79
Sex (male, %)	36, 48.60%
Eye (right, %)	73, 50.00%
Systemic medical history (positive, *n*, %)	10, 13.51%
Ocular comorbidity (positive, *n*, %)	37, 50.00%

**Table:** 

	Right eyes	**Left eyes**	***P*** **-value**
Axial length (mm)	24.29 (22.99, 26.26)	24.23 (23.03, 25.97)	0.799
K1 (D)	43.16 ± 2.02	43.15 ± 1.90	0.965
K1 axis (°)	95 (48, 151)	80 (27,118)	0.152
K2 (D)	44.34 ± 1.95	44.14 ± 1.93	0.538
K2 axis (°)	88.84 ± 52.56	92.75 ± 53.05	0.655
Corneal astigmatism (D)	−0.92 (−1.48, −0.49)	−0.64 (−1.12, −0.41)	0.165
Anterior chamber depth (mm)	3.19 ± 0.70	3.11 ± 0.64	0.452
Lens thickness (mm)	4.35 (4.00, 4.83)	4.41 (4.07, 4.74)	0.638
White-to-white (mm)	11.80 (11.50, 12.10)	11.81 ± 0.53	0.918
Chord μ (mm)	0.26 (0.20, 0.38)	0.21 (0.13, 0.31)	**0.026** ^*^
Chord α (mm)	0.40 (0.29, 0.50)	0.32 (0.26, 0.43)	**0.018** ^*^
B/F ratio (%)	82.00% (81.00%, 83.00%)	81.79% (80.80%, 83.59%)	0.608
Pupil diameter (mm)	3.46 (3.08, 4.47)	3.34 (2.79, 3.82)	0.069
Total corneal HOA (μm)	0.21 (0.15, 0.29)	0.23 ± 0.17	0.369
Total corneal Z40 (μm)	0.30 ± 0.18	0.29 ± 0.15	0.801
UCVA	0.35 (0.10, 0.60)	0.35 (0.10, 0.60)	1.000
UCVA (LogMAR)	0.46 (0.22, 1.00)	0.46 (0.22, 1.00)	1.000
BCVA	0.44 ± 0.33	0.44 ± 0.33	1.000
BCVA (LogMAR)	0.39 (0.22, 0.82)	0.39 (0.22, 0.82)	1.000

Angle Kappa and angle Alpha are, respectively, represented by chord μ and chord α in the Scheimpflug cameras (Pentacam, Oculus Optikgeräte GmbH, Wetzlar, Germany); BCVA, best corrected visual acuity; B/F ratio, the mean radius of the posterior corneal surface to the mean radius of the anterior corneal surface; D, diopter; Log MAR, logarithm of the minimum angle of resolution; K1, flattest keratometry; K2, steepest keratometry; Total corneal HOA, the total corneal higher-order aberrations calculated within the 4.0 mm zone around the corneal apex; Total corneal Z40, the total corneal spherical aberrations within the 6.0 mm zone around the corneal apex; UCVA, uncorrected visual acuity; ^*^P < 0.05. Bold values represent statistical significance (*P* < 0.05).

Of these surgeons, six were male and seven were female, with a statistically significant difference in sex distribution between high- and low-volume groups (*P* = 0.021). No significant differences in age, professional title, or education background were observed between the two groups (Table 2).

**Table 2:** Basic information and related surgical volume data of participating surgeons.

Variable	Total surgeons	High-volume surgeons	Low-volume surgeons	*P*-value
Number (*n*, %)	13 (100%)	8 (61.54%)	5 (38.46%)	/
Sex (male, %)	6 (46.20%)	6 (75.00%)	0 (0.00%)	**0.021** ^*^
Age (years)	41.54 ± 8.01	43.63 ± 9.01	38.20 ± 5.26	0.251
Surgical volume (cases/year)	403.00 ± 340.00	594.45 ± 290.83	96.50 ± 88.52	**0.004** ^*^
Professional title				0.604
Chief physician	4 (30.77%)	3 (37.50%)	1 (20.00%)	
Associate chief physician	6 (46.15%)	4 (50.00%)	2 (40.00%)	
Attending physician	3 (23.08%)	1 (12.50%)	2 (40.00%)	
Education background				1.000
Doctor	3 (23.08%)	2 (25.00%)	1 (20.00%)	
Master	7 (53.84%)	4 (50.00%)	3 (60.00%)	
Bachelor	3 (23.08%)	2 (25.00%)	1 (20.00%)	

Comparison between high-volume and low-volume surgeons, ^*^P < 0.05. Bold values represent statistical significance (*P* < 0.05).

### 3.2 Comparison of appropriateness

**Figure 2:**
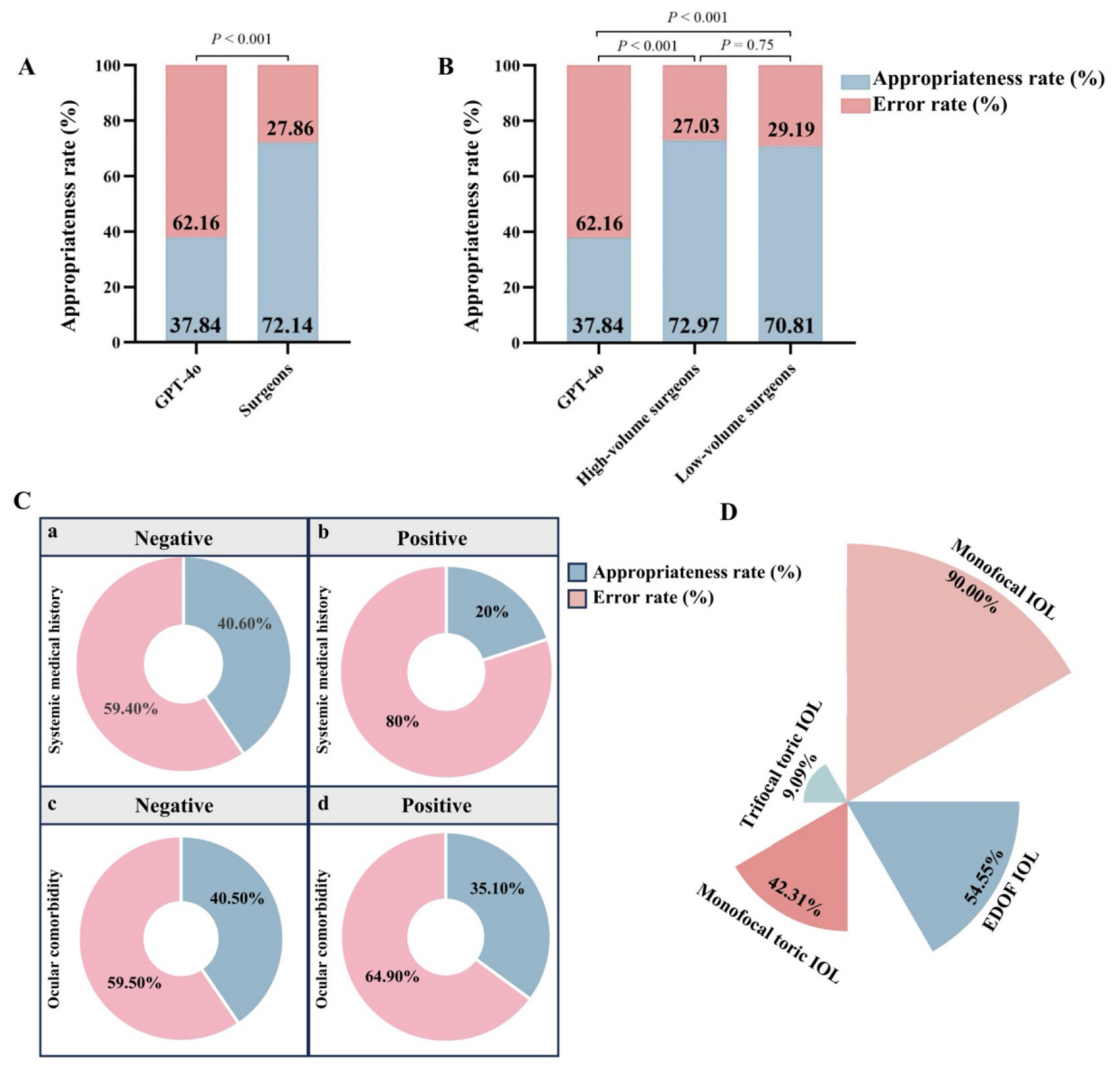
Comparison of appropriateness rates in IOL selection between GPT-4o and surgeons. **(A)** Appropriateness rate comparison between GPT-4o and all 13 surgeons. **(B)** Appropriateness rate comparison between GPT-4o and high-volume surgeons, low-volume surgeons. **(C)** GPT-4o appropriateness rates under different patient conditions: **(a)** Negative systemic medical history. **(b)** Positive systemic medical history. **(c)** Negative ocular comorbidity. **(d)** Positive ocular comorbidity. **(D)** GPT-4o appropriateness rates across different IOL types. IOL, intraocular lens.

The appropriateness rate of IOL selection by GPT-4o was significantly lower than that of surgeons (37.84% vs. 72.14%, *P* = 0.001; Figure 2A). Further comparison showed that high-volume surgeons had the highest appropriateness (72.97%), followed by low-volume surgeons (70.81%), whereas GPT-4o demonstrated the lowest alignment with expert consensus (*P* < 0.001; Figure 2B).

**Figure 3:**
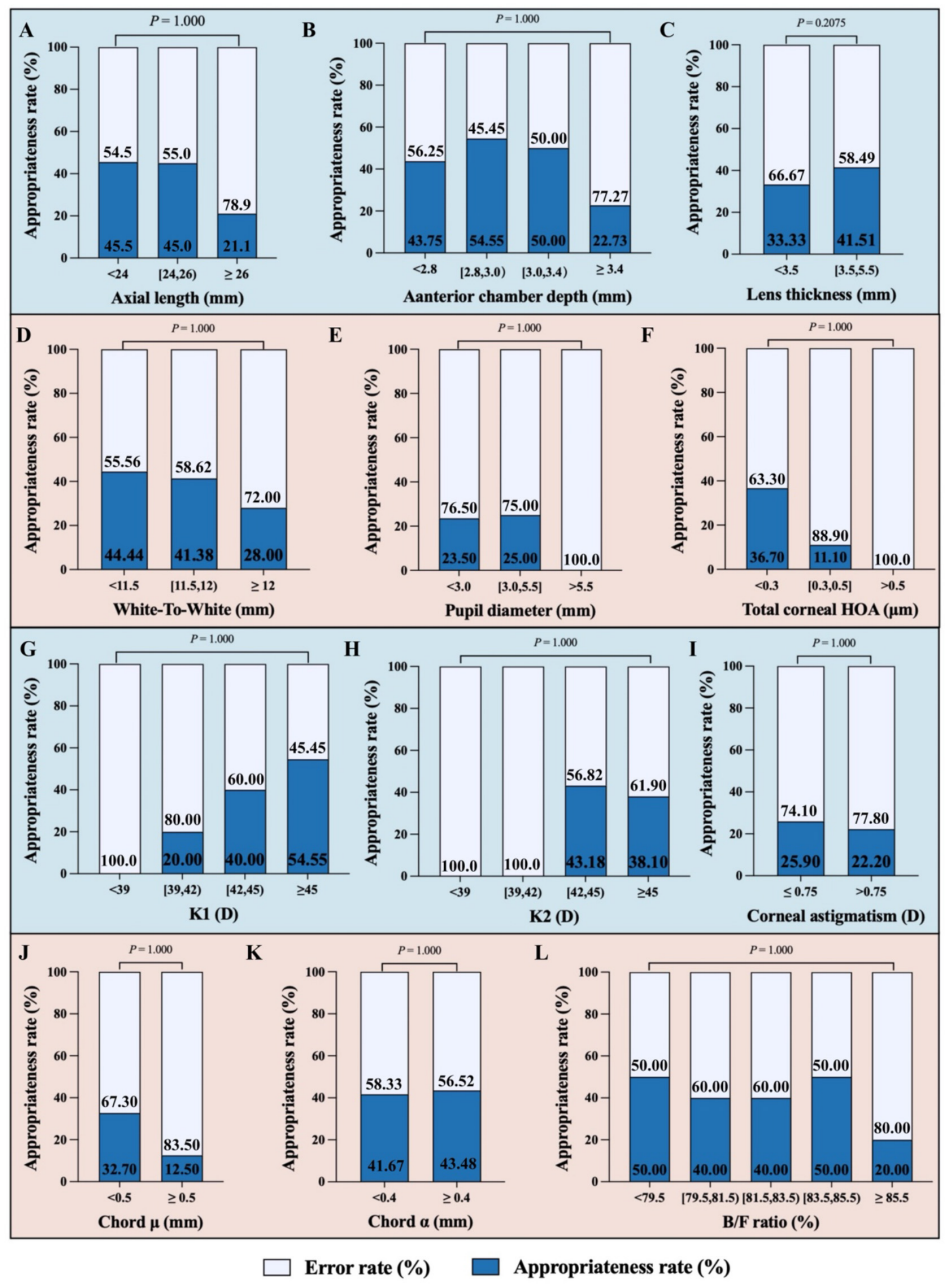
Comparison of GPT-4o appropriateness rates across different ocular parameters. **(A)** Axial lengths. **(B)** Anterior chamber depth. **(C)** Lens thickness. **(D)** White-to-white. **(E)** Pupil diameter. **(F)** Total corneal high-order aberrations calculated in the 4.0 mm zone around the corneal apex (Total corneal HOA). **(G)** Flattest keratometry (K1). **(H)** Steepest keratometry (K2). **(I)** Corneal astigmatism. **(J)** Chord μ (angle Kappa). **(K)** Chord α (angle Alpha). **(L)** B/F ratios (mean radius of the posterior corneal surface to the mean radius of the anterior corneal surface). D, diopter.

There were no statistically significant differences in GPT-4o's appropriateness rates between patients with and without a systemic clinical history (*P* = 1.000) or between those with and without ocular comorbidities (*P* = 1.000; Figure 2C). However, GPT-4o's appropriateness varied significantly across IOL types (*P* < 0.001), achieving the highest rate for monofocal IOLs (90.0%) and the lowest for trifocal toric IOLs (9.09%; Figure 2D). No significant differences were observed in GPT-4o's appropriateness across different ocular parameters (Figure 3).

Significant differences in IOL distribution were observed between GPT-4o and surgeons (*P* < 0.001). Surgeons were more likely to choose monofocal IOLs, whereas GPT-4o tended to select monofocal toric and trifocal toric IOLs (*P* < 0.001). For monofocal IOLs, surgeons demonstrated a more pronounced preference (*P* < 0.001), whereas GPT-4o exhibited stronger preferences for monofocal toric and trifocal toric IOLs (*P* < 0.001 for both). For bifocal and bifocal toric IOLs, the proportions selected by surgeons were higher than those chosen by GPT-4o (*P* = 0.006 and *P* = 0.043, respectively). When comparing IOL selection between GPT-4o and high-volume vs. low-volume surgeons, GPT-4o showed a more pronounced tendency to select EDOF IOLs and a lower inclination toward trifocal IOLs (*P* = 0.005, *P* < 0.001, respectively; Table 3).

**Table 3:** Analysis of the distribution and consistency of IOL selection by GPT-4o and different groups of surgeons.

Variable	GPT-4o	Surgeons	*P*-value^#^	High-volume surgeons	Low-volume surgeons	*P*-value^##^
Monofocal IOL	13.51%	52.31%	**< 0.001** ^*^	52.55%	51.94%	**< 0.001** ^*^
Bifocal IOL	0.00%	8.83%	**0.006** ^*^	8.79%	8.89%	**0.023** ^*^
Extended depth of focus IOL	14.86%	10.23%	0.148	11.60%	8.06%	**0.005** ^*^
Trifocal IOL	6.76%	11.63%	0.254	8.61%	16.39%	**< 0.001** ^*^
Monofocal toric IOL	35.14%	8.61%	**< 0.001** ^*^	10.02%	6.39%	**< 0.001** ^*^
Bifocal toric IOL	0.00%	4.41%	**0.043** ^*^	4.57%	4.17%	0.102
Trifocal toric IOL	29.73%	3.98%	**< 0.001** ^*^	3.87%	4.17%	**< 0.001** ^*^
Intraclass correlation coefficient	0.378	0.359	/	0.399	0.247	/
Kappa value (with GPT-4o)	/	0.038^**^	/	0.096^**^	0.038^**^	/

IOL, intraocular lens; ^#^Chi-square comparison between GPT-4o and total surgeons; ^*##*^Chi-square comparison between GPT-4o, high-volume, and low-volume surgeons. ^*^P < 0.05, ^**^indicates the *P*-value corresponding to the Kappa value is < 0.05. Bold values represent statistical significance (*P* < 0.05).

**Figure 4:**
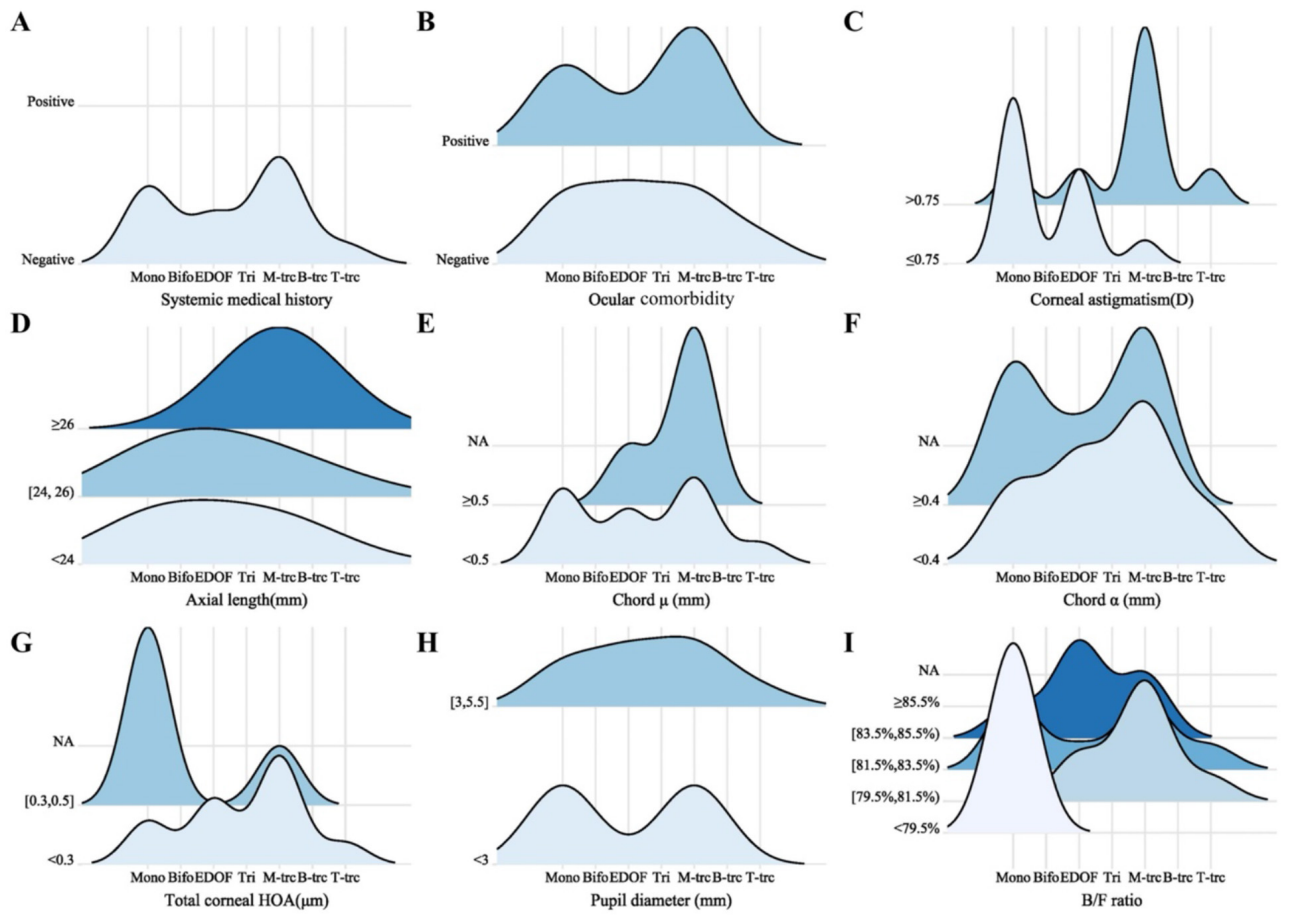
Distribution of different IOL types selected by GPT-4o under various conditions and ocular parameters. **(A)** Presence or absence of systemic medical history. **(B)** Presence or absence of ocular comorbidity. **(C)** Different levels of corneal astigmatism. **(D)** Different axial lengths. **(E)** Different chord μ (angle Kappa). **(F)** Different chord α (angle Alpha). **(G)** Different total corneal high-order aberrations calculated in the 4.0 mm zone around the corneal apex (Total corneal HOA). **(H)** Different pupil diameter. **(I)** Different B/F ratios (mean radius of the posterior corneal surface to the mean radius of the anterior corneal surface). Mono, Monofocal IOL; Bifo, Bifocal IOL; EDOF, Extended depth of focus IOL; Tri, Trifocal IOL; M-trc, Monofocal toric IOL; B-trc, Bifocal toric IOL; T-trc, Trifocal toric IOL; D, diopter; IOL, intraocular lens.

The main types of IOL selection by GPT-4o varied across clinical scenarios. When ocular comorbidities were present, GPT-4o primarily selected monofocal and monofocal toric IOLs, whereas a broader range of selections were observed in cases without comorbidities. For corneal astigmatism ≤ 0.75D, GPT-4o favored monofocal and EDOF IOLs, while for corneal astigmatism > 0.75D, monofocal toric IOLs were predominantly chosen. A preference for monofocal toric IOLs was also observed when Chord μ was ≥0.5 mm or Chord α was ≥0.4 mm. For total corneal HOA between 0.3 and 0.5 μm, GPT-4o focused on monofocal and monofocal toric IOLs. Pupil diameter influenced selection as well: for pupil diameter < 3 mm, monofocal and monofocal toric IOLs were preferred. When the B/F ratio was < 79.5%, GPT-4o exclusively selected monofocal IOLs (Figure 4).

### 3.3 Consistency analysis

**Figure 5:**
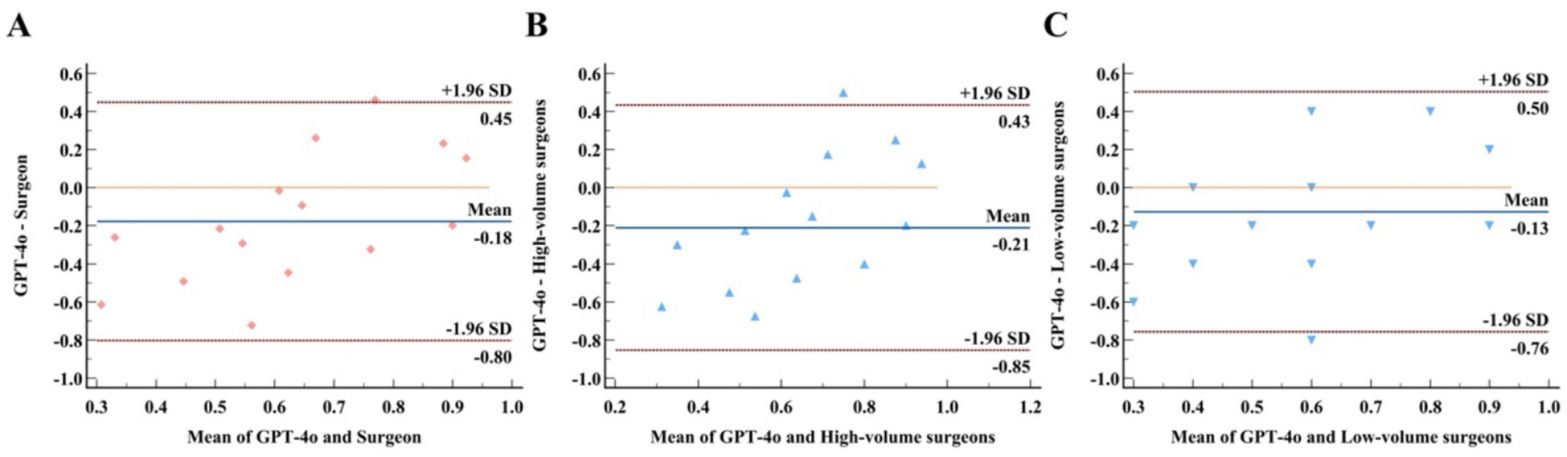
Comparison of consistency between GPT-4o appropriateness over five repetitions and appropriateness of surgeons. **(A)** GPT-4o vs. all surgeons. **(B)** GPT-4o vs. high-volume surgeons. **(C)** GPT-4o vs. low-volume surgeons. SD, standard deviation.

The ICC of GPT-4o (0.378) was slightly higher than that of surgeons (0.359), but both demonstrated generally low consistency. Agreement analysis using Kappa values showed that GPT-4o consistency with high-volume surgeons (Kappa = 0.096) exceeded that with all surgeons or low-volume surgeons (Kappa = 0.038 for both), yet overall agreement remained poor (Kappa < 0.4 for all comparisons; Table 3). Bland–Altman analysis indicated limits of agreement of −0.80 to 0.45 between GPT-4o and all surgeons (mean = −0.18), −0.85 to 0.43 for GPT-4o vs. high-volume surgeons (mean = −0.21), and −0.76 to −0.13 for GPT-4o vs. low-volume surgeons (mean = −0.76; Figure 5). Inter-surgeon agreement was slight, with Kappa values of 0.196 (95% CI: 0.173–0.219) for high-volume surgeons, 0.136 (95% CI: 0.100–0.173) for low-volume surgeons, and 0.194 (95% CI: 0.181–0.207) for the entire cohort, reflecting the inherent variability in complex IOL decision-making.

## 4 Discussion

Determining the most appropriate IOL for each patient remains a complex and clinically critical task (Alio et al., 2017; Niazi et al., 2024; de Vries et al., 2011; Gibbons et al., 2016; Hall et al., 2022). This study provides one of the first real-world evaluations of GPT-4o in IOL selection, comparing its decision-making process with that of experienced cataract surgeons (Lundstrom et al., 2018; Yeu and Cuozzo, 2021). Our findings indicate that GPT-4o achieves high appropriateness with expert consensus for monofocal IOL selection but shows reduced performance for functional lenses, including multifocal and toric designs. These results underscore both the potential and the current limitations of LLMs in ophthalmology (Oustoglou et al., 2023). GPT-4o may serve as a supportive reference for final surgical decisions or as a guidance tool for junior clinicians and residents; however, its recommendations should be interpreted cautiously (Mitsuyama et al., 2025).

Across surgeons with varying levels of experience, GPT-4o consistently demonstrated lower appropriateness than human surgeons. The presence of systemic or ocular comorbidities further reduced model performance, indicating limited flexibility in handling complex clinical factors (Xiong et al., 2025). In these scenarios, the model tended to favor more conservative strategies, typically recommending monofocal or monofocal toric IOLs. Specifically, GPT-4o achieved a high appropriateness rate (90.0%) for monofocal IOL selection, whereas its performance was notably weaker for functional lenses, such as trifocal or multifocal toric IOLs. This discrepancy likely reflects the relatively straightforward nature of monofocal IOL selection compared with the nuanced, multidimensional decision-making required for functional IOLs. The absence of bifocal IOL selections likely reflects a specific weighting of clinical priorities within the model's training data rather than an inherent lack of capability. Although variations in ocular parameters had minimal impact on overall performance, appropriateness fell below average in cases with extreme biometric ranges. Notably, surgeons' preference for monofocal IOLs contrasted with GPT-4o's inclination toward advanced types such as trifocal toric lenses, highlighting a divergence in clinical strategies between AI and human practitioners.

Comparative analyses revealed that GPT-4o exhibited a distinct selection pattern. Its stability across clinical scenarios was lower than that of human surgeons (Strong et al., 2023; Gomez-Cabello et al., 2024). Moreover, the higher agreement between GPT-4o and high-volume surgeons than with low-volume surgeons suggests that the model partially captures expert-like decision patterns, although the overall agreement remained poor. Similarly, the relatively low inter-surgeon agreement (Kappa 0.136–0.196) underscores that IOL selection is inherently a “preference-sensitive task” rather than a standardized process with a single optimal outcome (Zamora-de La Cruz et al., 2023). Consequently, the primary value of LLMs in this context is not to replace human intuition but to provide a guideline-based framework to reduce non-rational variability and support complex decisions (Shi et al., 2025). By aligning with a consensus-based reference, LLMs can function as a consistent “second opinion” to bridge divergent clinical perspectives (Horiuchi et al., 2024; Huang et al., 2024; Noda et al., 2025).

Analysis of raw outputs demonstrated that GPT-4o applies clear reasoning chains, particularly in cases with complex visual demands. For example, in a patient with −2.28 D corneal astigmatism and high expectations for spectacle independence, the model systematically prioritized trifocal technology to cover the visual range. It specifically recommended a toric variant to correct astigmatism while balancing the goal of spectacle independence against potential postoperative disturbances such as glare and halos.

These observations indicate that GPT-4o can integrate objective ocular parameters with patient-specific quality-of-life considerations within a discernible clinical framework. However, the model exhibits a tendency to favor aggressive interventions to meet subjective patient expectations while potentially underweighting principles of clinical robustness. Our results align with recent literature, suggesting that GPT-4o's performance in complex scenarios does not yet reach expert ophthalmologist levels (Gomez-Cabello et al., 2024; Horiuchi et al., 2024). To minimize clinical risk, LLM-based recommendations must be applied judiciously to prevent errors such as hallucinations or logical inconsistencies (Xiong et al., 2025; Shi et al., 2025). Cai et al. reported that LLMs, including GPT-4o, underperform in medical imaging interpretation, a critical component of advanced ophthalmic care (Cai et al., 2023). Similarly, Ali et al. emphasized that LLMs often produce confidently incorrect responses for complex queries without standardized answers, highlighting significant risks for clinical deployment (Ali et al., 2023). Collectively, these findings indicate that while LLMs show promise for basic examinations, they remain insufficient for tasks requiring high-level clinical judgment (Anguita et al., 2024; Bibault et al., 2019).

In specific comparative studies, Stremmel et al. found that when diagnostic outcomes are clear and unique, GPT-4 outperforms GPT-3.5 and emergency residents (Wen et al., 2025; Hoppe et al., 2024). Noda et al. demonstrated that GPT-4o offers significant advantages as a “decision support tool” rather than as an “independent decision-maker,” enhancing clinician performance (Noda et al., 2025). The current challenge is to integrate GPT-4o effectively into clinical workflows as a diagnostic aid (Wen et al., 2025). Existing evidence suggests that LLMs can reduce cognitive load, simulate structured reasoning, and assist with immediate tasks; thus, their optimal role is to augment rather than replace human expertise (Gomez-Cabello et al., 2024; Ayers et al., 2023; Goodman et al., 2023; Jung et al., 2025).

This study has several limitations. First, its single-center design and relatively small sample size may limit generalizability. Second, categorizing IOLs into seven types resulted in small numbers in some subgroups, which limited statistical power and the reliability of subgroup analyses. Third, because LLMs are rapidly updated, GPT-4o performance may vary across versions; therefore, our findings should be interpreted as a time-bound snapshot. Finally, this study evaluated only GPT-4o. Future multicenter studies with larger datasets should include continuous version tracking, interpretability analyses, and comparisons with other LLMs, such as Claude and Gemini.

## 5 Conclusion

GPT-4o demonstrated reliable appropriateness in selecting standard monofocal IOLs but insufficient performance in complex or multifocal cases. Although the model shows potential for supporting routine clinical decision-making, further refinement with ophthalmology-specific data and multimodal integration is essential before clinical adoption. LLMs should currently be viewed as assistive, not autonomous, tools for cataract surgery planning.

## Data Availability

The original contributions presented in the study are included in the article/Supplementary material, further inquiries can be directed to the corresponding author/s.

## References

[B1] Ali R. Tang O. Y. Connolly I. D. Fridley J. S. Shin J. H. Zadnik Sullivan P. L. . (2023). Performance of ChatGPT, GPT-4, and Google bard on a neurosurgery oral boards preparation question bank. Neurosurgery 93, 1090–1098. doi: 10.1227/neu.0000000000002551 37306460

[B2] Alio J. L. Plaza-Puche A. B. Fernandez-Buenaga R. Pikkel J. Maldonado M. (2017). Multifocal intraocular lenses: an overview. Surv. Ophthalmol. 62, 611–634. doi: 10.1016/j.survophthal.2017.03.005 28366683

[B3] Anguita R. Downie C. Ferro Desideri L. Sagoo M. S. (2024). Assessing large language models' accuracy in providing patient support for choroidal melanoma. Eye (Lond.) 38, 3113–3117. doi: 10.1038/s41433-024-03231-w 39003430 PMC11544095

[B4] Antaki F. Touma S. Milad D. El-Khoury J. Duval R. (2023). Evaluating the performance of ChatGPT in ophthalmology: an analysis of its successes and shortcomings. Ophthalmol. Sci. 3:100324. doi: 10.1016/j.xops.2023.100324 37334036 PMC10272508

[B5] Ayers J. W. Poliak A. Dredze M. Leas E. C. Zhu Z. Kelley J. B. . (2023). Comparing physician and artificial intelligence chatbot responses to patient questions posted to a public social media forum. JAMA Intern. Med. 183, 589–596. doi: 10.1001/jamainternmed.2023.1838 37115527 PMC10148230

[B6] Bernstein I. A. Zhang Y. V. Govil D. Majid I. Chang R. T. Sun Y. . (2023). Comparison of ophthalmologist and large language model chatbot responses to online patient eye care questions. JAMA Netw. Open 6:e2330320. doi: 10.1001/jamanetworkopen.2023.30320 37606922 PMC10445188

[B7] Betzler B. K. Chen H. Cheng C. Y. Lee C. S. Ning G. Song S. J. . (2023). Large language models and their impact in ophthalmology. Lancet Digit. Health 5, e917–e924. doi: 10.1016/S2589-7500(23)00201-7 38000875 PMC11003328

[B8] Bibault J. E. Chaix B. Guillemasse A. Cousin S. Escande A. Perrin M. . (2019). A chatbot versus physicians to provide information for patients with breast cancer: blind, randomized controlled noninferiority trial. J. Med. Internet Res. 21:e15787. doi: 10.2196/15787 31774408 PMC6906616

[B9] Cai L. Z. Shaheen A. Jin A. Fukui R. Yi J. S. Yannuzzi N. . (2023). Performance of generative large language models on ophthalmology board–style questions. Am. J. Ophthalmol. 254, 141–149. doi: 10.1016/j.ajo.2023.05.024 37339728

[B10] Chen J. S. Reddy A. J. Al-Sharif E. Shoji M. K. Kalaw F. G. P. Eslani M. . (2025). Analysis of ChatGPT responses to ophthalmic cases: can ChatGPT think like an ophthalmologist? Ophthalmol. Sci. 5:100600. doi: 10.1016/j.xops.2024.100600 39346575 PMC11437840

[B11] Clusmann J. Kolbinger F. R. Muti H. S. Carrero Z. I. Eckardt J. N. Laleh N. G. . (2023). The future landscape of large language models in medicine. Commun. Med. (Lond.) 3:141. doi: 10.1038/s43856-023-00370-1 37816837 PMC10564921

[B12] Coassin M. Di Zazzo A. Antonini M. Gaudenzi D. Gallo Afflitto G. Kohnen T. . (2020). Extended depth-of-focus intraocular lenses: power calculation and outcomes. J. Cataract Refract. Surg. 46, 1554–1560. doi: 10.1097/j.jcrs.0000000000000293 32590481

[B13] de Vries N. E. Webers C. A. Touwslager W. R. Bauer N. J. de Brabander J. Berendschot T. T. . (2011). Dissatisfaction after implantation of multifocal intraocular lenses. J. Cataract Refract. Surg. 37, 859–865. doi: 10.1016/j.jcrs.2010.11.032 21397457

[B14] Gibbons A. Ali T. K. Waren D. P. Donaldson K. E. (2016). Causes and correction of dissatisfaction after implantation of presbyopia-correcting intraocular lenses. Clin. Ophthalmol. 10, 1965–1970. doi: 10.2147/OPTH.S114890 27784985 PMC5066995

[B15] Gomez-Cabello C. A. Borna S. Pressman S. M. Haider S. A. Forte A. J. (2024). Large language models for intraoperative decision support in plastic surgery: a comparison between ChatGPT-4 and Gemini. Medicina (Kaunas) 60:957. doi: 10.3390/medicina60060957 38929573 PMC11205293

[B16] Goodman R. S. Patrinely J. R. Stone C. A. Jr. Zimmerman E. Donald R. R. Chang S. S. . (2023). Accuracy and reliability of chatbot responses to physician questions. JAMA Netw. Open 6:e2336483. doi: 10.1001/jamanetworkopen.2023.36483 37782499 PMC10546234

[B17] Hall C. D. Barnes C. S. Gutherie A. H. Lynch M. G. (2022). Visual function and mobility after multifocal versus monofocal intraocular lens implantation. Clin. Exp. Optom. 105, 70–76. doi: 10.1080/08164622.2021.1896337 33730524

[B18] Hoppe J. M. Auer M. K. Struven A. Massberg S. Stremmel C. (2024). ChatGPT with GPT-4 outperforms emergency department physicians in diagnostic accuracy: retrospective analysis. J. Med. Internet Res. 26:e56110. doi: 10.2196/56110 38976865 PMC11263899

[B19] Horiuchi D. Tatekawa H. Oura T. Oue S. Walston S. L. Takita H. . (2024). Comparing the diagnostic performance of GPT-4-based ChatGPT, GPT-4V-based ChatGPT, and radiologists in challenging neuroradiology cases. Clin. Neuroradiol. 34, 779–787. doi: 10.1007/s00062-024-01426-y 38806794

[B20] Huang X. Estau D. Liu X. Yu Y. Qin J. Li Z. (2024). Evaluating the performance of ChatGPT in clinical pharmacy: a comparative study of ChatGPT and clinical pharmacists. Br. J. Clin. Pharmacol. 90, 232–238. doi: 10.1111/bcp.15896 37626010

[B21] Jung H. Oh J. Stephenson K. A. J. Joe A. W. Mammo Z. N. (2025). Prompt engineering with ChatGPT3.5 and GPT4 to improve patient education on retinal diseases. Can. J. Ophthalmol. 60, e375–e381. doi: 10.1016/j.jcjo.2024.08.010 39245293

[B22] Kohnen T. (2019). Questionnaires for cataract and refractive surgery. J. Cataract Refract. Surg. 45, 119–120. doi: 10.1016/j.jcrs.2018.12.020 30704727

[B23] Lim Z. W. Pushpanathan K. Yew S. M. E. Lai Y. Sun C. H. Lam J. S. H. . (2023). Benchmarking large language models' performances for myopia care: a comparative analysis of ChatGPT-3.5, ChatGPT-4.0, and Google Bard. EBioMedicine 95:104770. doi: 10.1016/j.ebiom.2023.104770 37625267 PMC10470220

[B24] Liu J. Wang C. Liu S. (2023). Utility of ChatGPT in clinical practice. J. Med. Internet Res. 25:e48568. doi: 10.2196/48568 37379067 PMC10365580

[B25] Logothetis H. D. Feder R. S. (2019). Which intraocular lens would ophthalmologists choose for themselves? Eye 33, 1635–1641. doi: 10.1038/s41433-019-0460-9 31089237 PMC7002714

[B26] Lundstrom M. Dickman M. Henry Y. Manning S. Rosen P. Tassignon M. J. . (2018). Risk factors for refractive error after cataract surgery: analysis of 282 811 cataract extractions reported to the European Registry of Quality Outcomes for cataract and refractive surgery. J. Cataract Refract. Surg. 44, 447–452. doi: 10.1016/j.jcrs.2018.01.031 29685779

[B27] Lyons R. J. Arepalli S. R. Fromal O. Choi J. D. Jain N. (2024). Artificial intelligence chatbot performance in triage of ophthalmic conditions. Can. J. Ophthalmol. 59, e301–e308. doi: 10.1016/j.jcjo.2023.07.016 37572695

[B28] Maywood M. J. Ravi P. Deobhakta A. Begaj T. (2024). Performance assessment of an artificial intelligence chatbot in clinical vitreoretinal scenarios. Retina 44, 954–964. doi: 10.1097/IAE.0000000000004053 38271674

[B29] Mitsuyama Y. Tatekawa H. Takita H. Sasaki F. Tashiro A. Oue S. . (2025). Comparative analysis of GPT-4-based ChatGPT's diagnostic performance with radiologists using real-world radiology reports of brain tumors. Eur. Radiol. 35, 1938–1947. doi: 10.1007/s00330-024-11032-8 39198333 PMC11913992

[B30] Niazi S. Gatzioufas Z. Dhubhghaill S. N. Moshirfar M. Faramarzi A. Mohammadi F. . (2024). Association of patient satisfaction with cataract grading in five types of multifocal IOLs. Adv. Ther. 41, 231–245. doi: 10.1007/s12325-023-02698-5 37884810

[B31] Noda R. Tanabe K. Ichikawa D. Shibagaki Y. (2025). GPT4′s performance in supporting physician decision-making in nephrology multiple-choice questions. Sci. Rep. 15:15439. doi: 10.1038/s41598-025-99774-3 40316716 PMC12048615

[B32] Oustoglou E. Tzamalis A. Banou L. Christou C. D. Tsinopoulos I. Samouilidou M. . (2023). When should cataract surgeons seek assistance from experienced colleagues? Int. Ophthalmol. 43, 387–395. doi: 10.1007/s10792-022-02434-y 35864285

[B33] Shi M. J. Wang Z. X. Wang S. K. Li X. H. Zhang Y. L. Yan Y. . (2025). Performance of GPT-4 for automated prostate biopsy decision-making based on mpMRI: a multi-center evidence study. Mil. Med. Res. 12:33. doi: 10.1186/s40779-025-00621-3 40619425 PMC12232764

[B34] Strong E. DiGiammarino A. Weng Y. Kumar A. Hosamani P. Hom J. . (2023). Chatbot vs medical student performance on free-response clinical reasoning examinations. JAMA Intern. Med. 183, 1028–1030. doi: 10.1001/jamainternmed.2023.2909 37459090 PMC10352923

[B35] Wen R. Li X. Chen K. Sun M. Zhu C. Xu P. . (2025). GPT-4 vs. radiologists: who advances mediastinal tumor classification better across report quality levels? A cohort study. Int. J. Surg. 111, 9000–9011. doi: 10.1097/JS9.0000000000003127 40788014 PMC12695208

[B36] Xiong Y. T. Lian W. J. Sun Y. N. Liu W. Guo J. X. Tang W. . (2025). Exploring GPT-4o's multimodal reasoning capabilities with panoramic radiograph: the role of prompt engineering. Clin. Oral Investig. 29:405. doi: 10.1007/s00784-025-06498-9 40790280

[B37] Yeu E. Cuozzo S. (2021). Matching the patient to the intraocular lens. Ophthalmology 128, e132–e141. doi: 10.1016/j.ophtha.2020.08.025 32882308

[B38] Zamora-de La Cruz D. Zuniga-Posselt K. Bartlett J. Gutierrez M. Abariga S. A. (2023). Trifocal intraocular lenses versus bifocal intraocular lenses after cataract extraction among participants with presbyopia. Cochrane Database Syst. Rev. 6:CD012648. doi: 10.1002/14651858.CD012648.pub3 32584432 PMC7388867

