# Supplementary figures and images for: Assessing GPT-4o in cataract surgery decision-making: appropriateness, consistency, and clinical implications
Source: Front Artif Intell. 2026 May 29;9:1810899. doi: 10.3389/frai.2026.1810899 (PMC13260408; doi:10.3389/frai.2026.1810899)

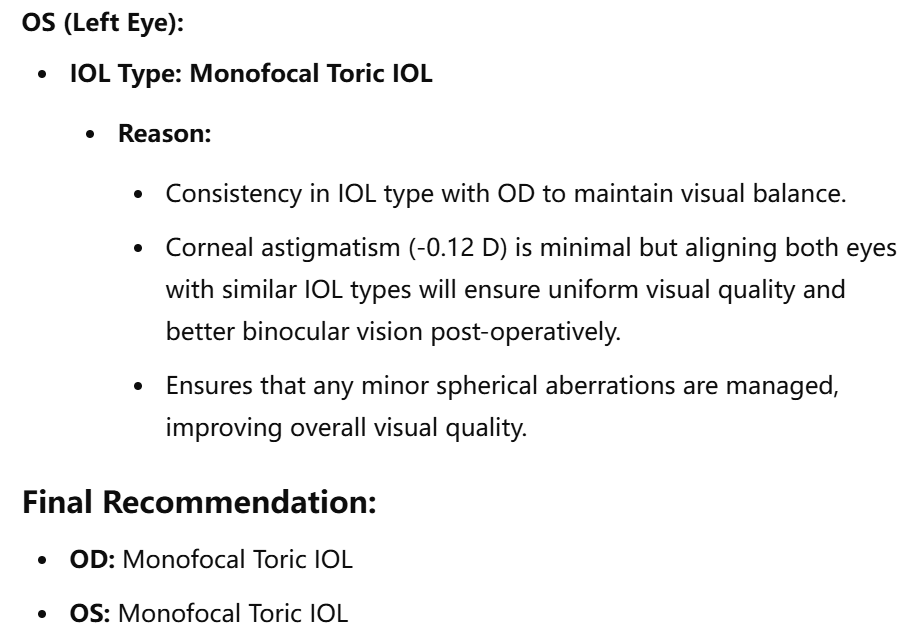

Supplement: Supplementary file 1 [file Data_Sheet_1.zip › supplementary material 3/95/σ▒Åσ╣òμê¬σ¢╛ 2024-08-09 064544.png]

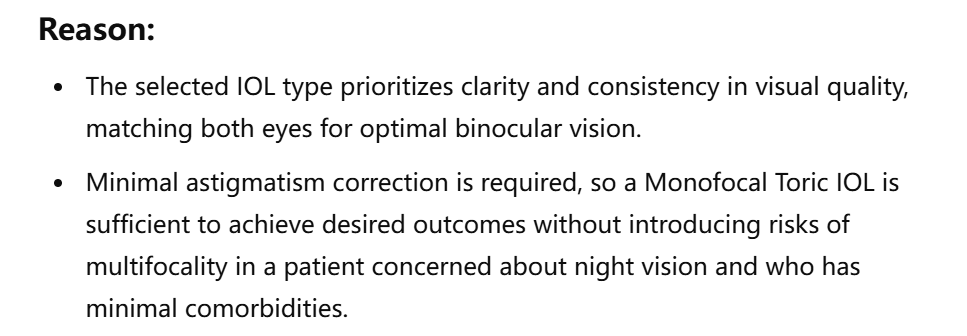

Supplement: Supplementary file 1 [file Data_Sheet_1.zip › supplementary material 3/95/σ▒Åσ╣òμê¬σ¢╛ 2024-08-09 064557.png]

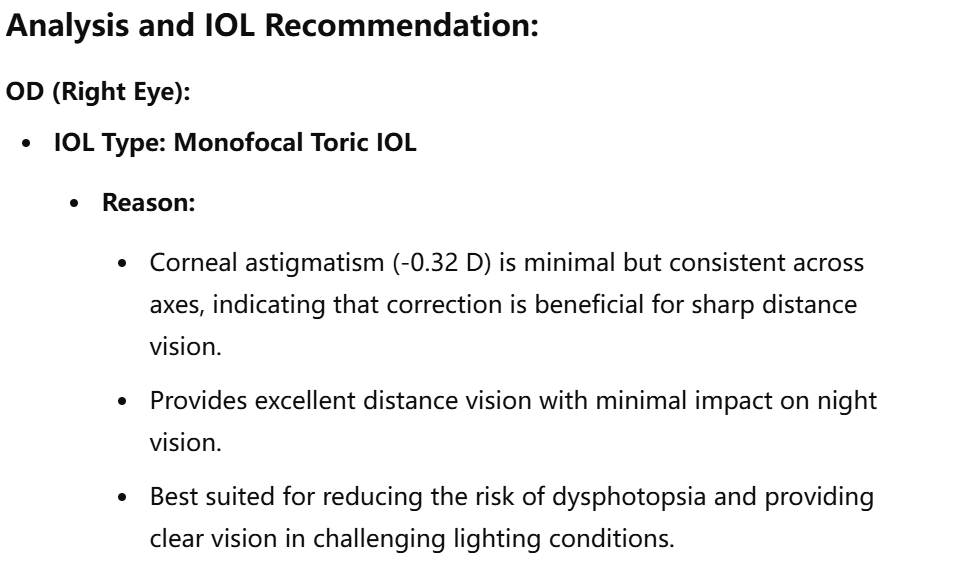

Supplement: Supplementary file 1 [file Data_Sheet_1.zip › supplementary material 3/95/σ▒Åσ╣òμê¬σ¢╛ 2024-08-09 064529.png]

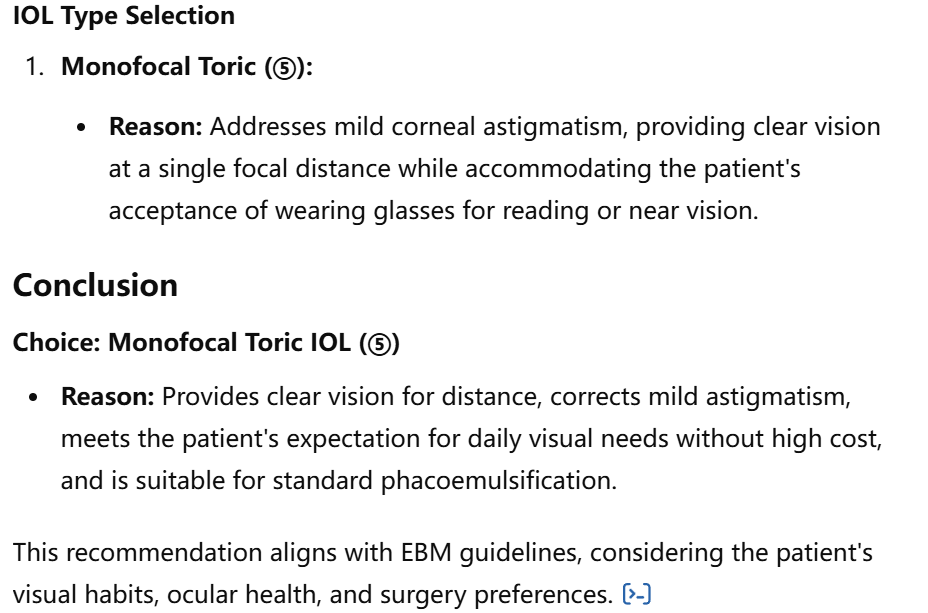

Supplement: Supplementary file 1 [file Data_Sheet_1.zip › supplementary material 3/132/σ▒Åσ╣òμê¬σ¢╛ 2024-08-06 020321.png]

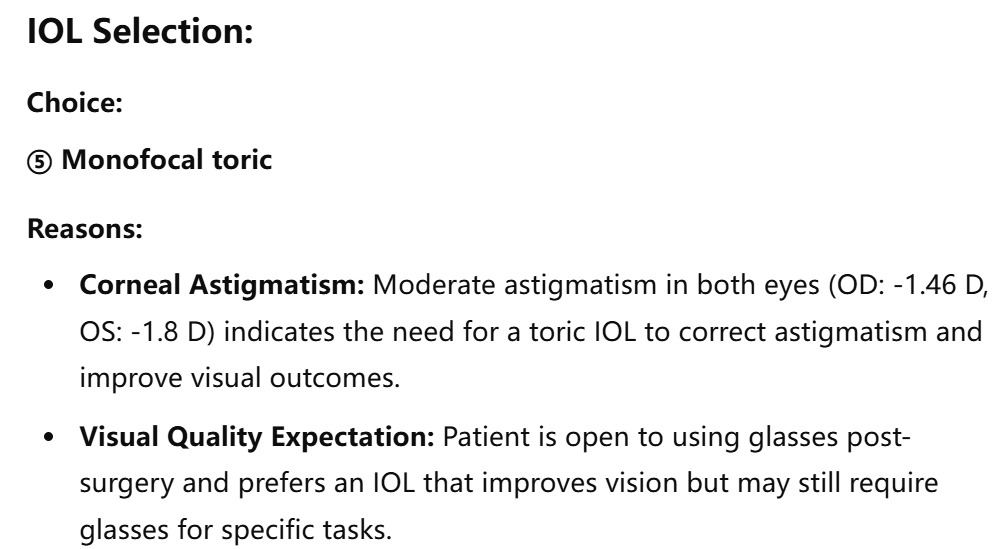

Supplement: Supplementary file 1 [file Data_Sheet_1.zip › supplementary material 3/104/σ▒Åσ╣òμê¬σ¢╛ 2024-08-09 132222.png]

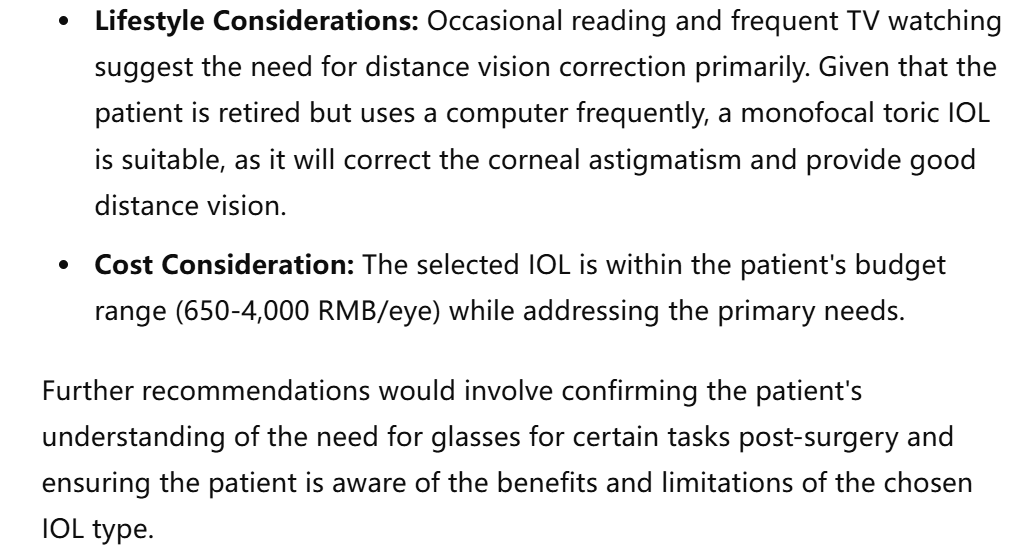

Supplement: Supplementary file 1 [file Data_Sheet_1.zip › supplementary material 3/104/σ▒Åσ╣òμê¬σ¢╛ 2024-08-09 132250.png]

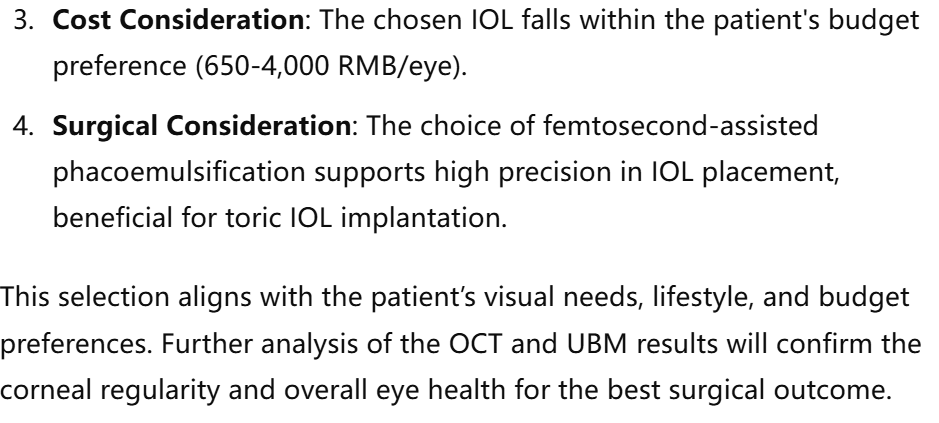

Supplement: Supplementary file 1 [file Data_Sheet_1.zip › supplementary material 3/102/σ▒Åσ╣òμê¬σ¢╛ 2024-08-09 065856.png]

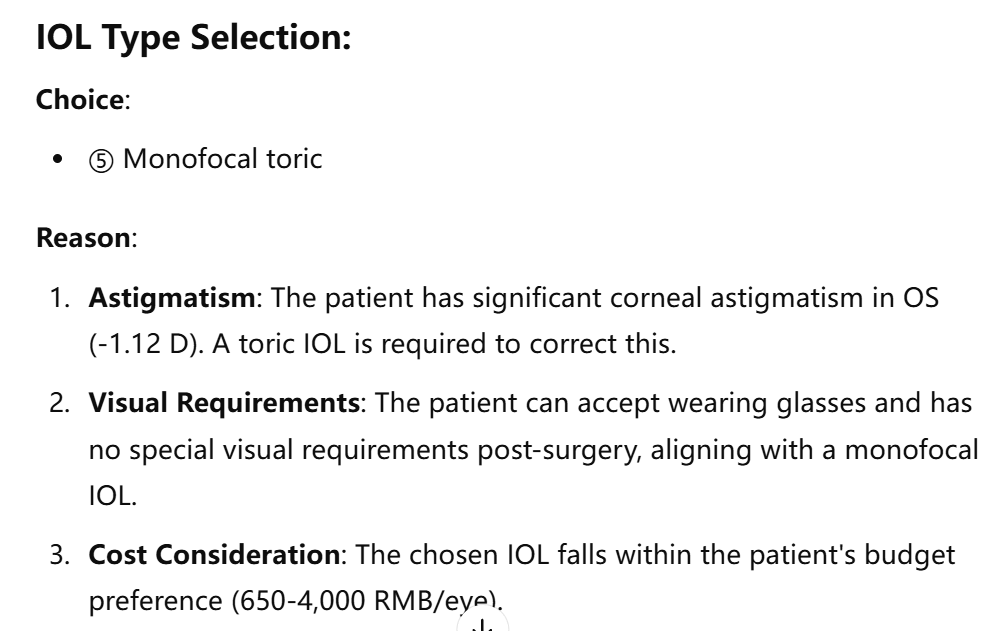

Supplement: Supplementary file 1 [file Data_Sheet_1.zip › supplementary material 3/102/σ▒Åσ╣òμê¬σ¢╛ 2024-08-09 065830.png]

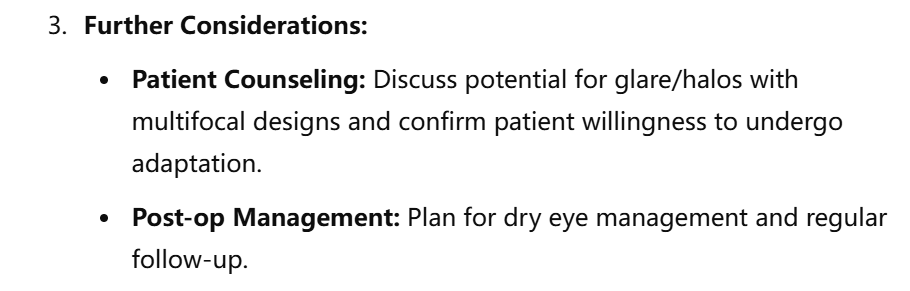

Supplement: Supplementary file 1 [file Data_Sheet_1.zip › supplementary material 3/93/σ▒Åσ╣òμê¬σ¢╛ 2024-08-09 064308.png]

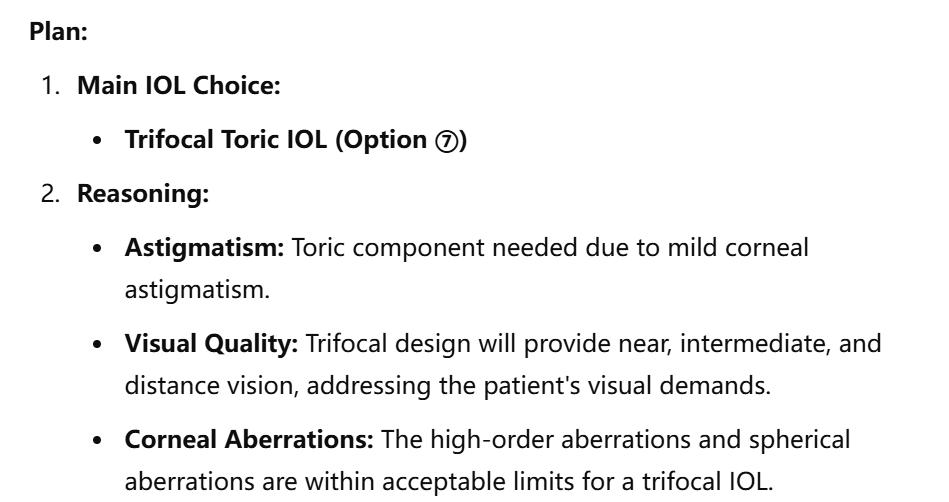

Supplement: Supplementary file 1 [file Data_Sheet_1.zip › supplementary material 3/93/σ▒Åσ╣òμê¬σ¢╛ 2024-08-09 064255.png]

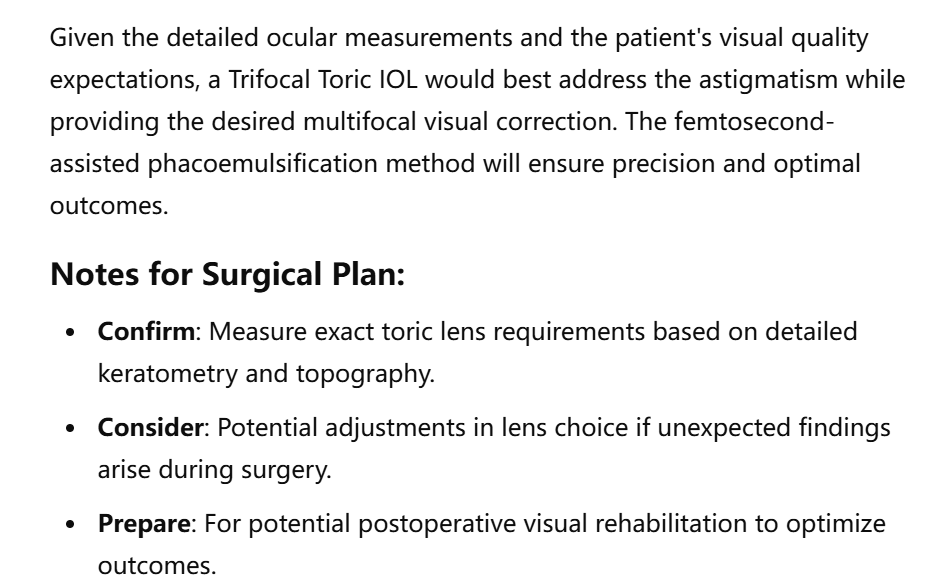

Supplement: Supplementary file 1 [file Data_Sheet_1.zip › supplementary material 3/134/σ▒Åσ╣òμê¬σ¢╛ 2024-08-06 020714.png]

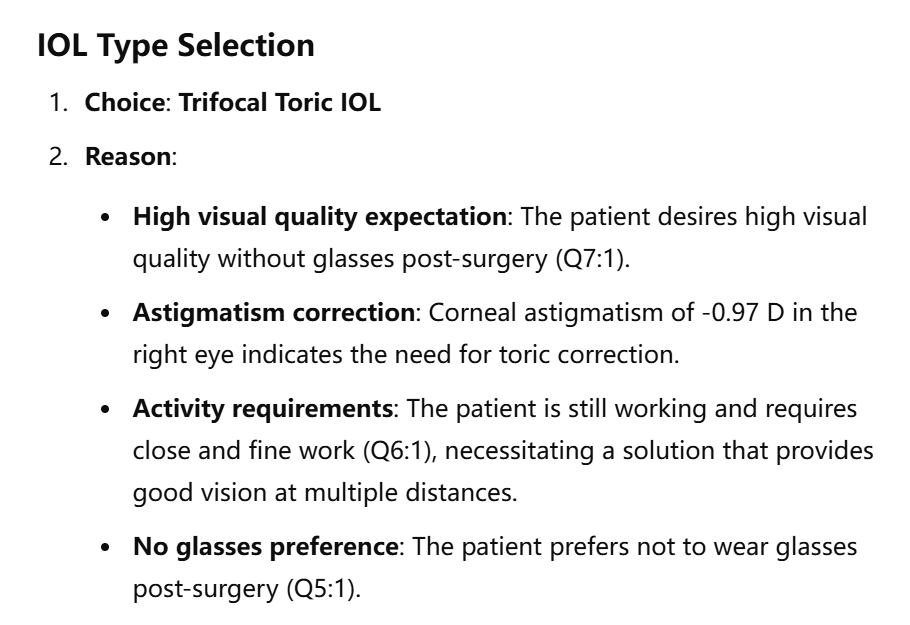

Supplement: Supplementary file 1 [file Data_Sheet_1.zip › supplementary material 3/134/σ▒Åσ╣òμê¬σ¢╛ 2024-08-06 020646.png]

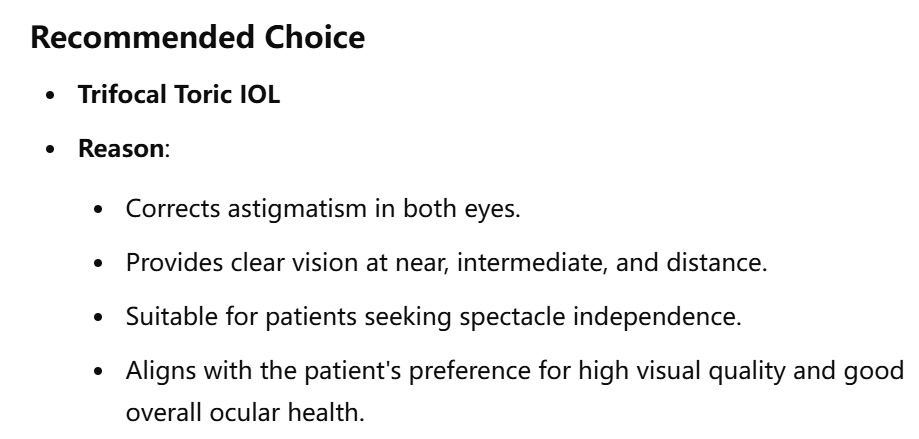

Supplement: Supplementary file 1 [file Data_Sheet_1.zip › supplementary material 3/142/σ▒Åσ╣òμê¬σ¢╛ 2024-08-06 023151.png]

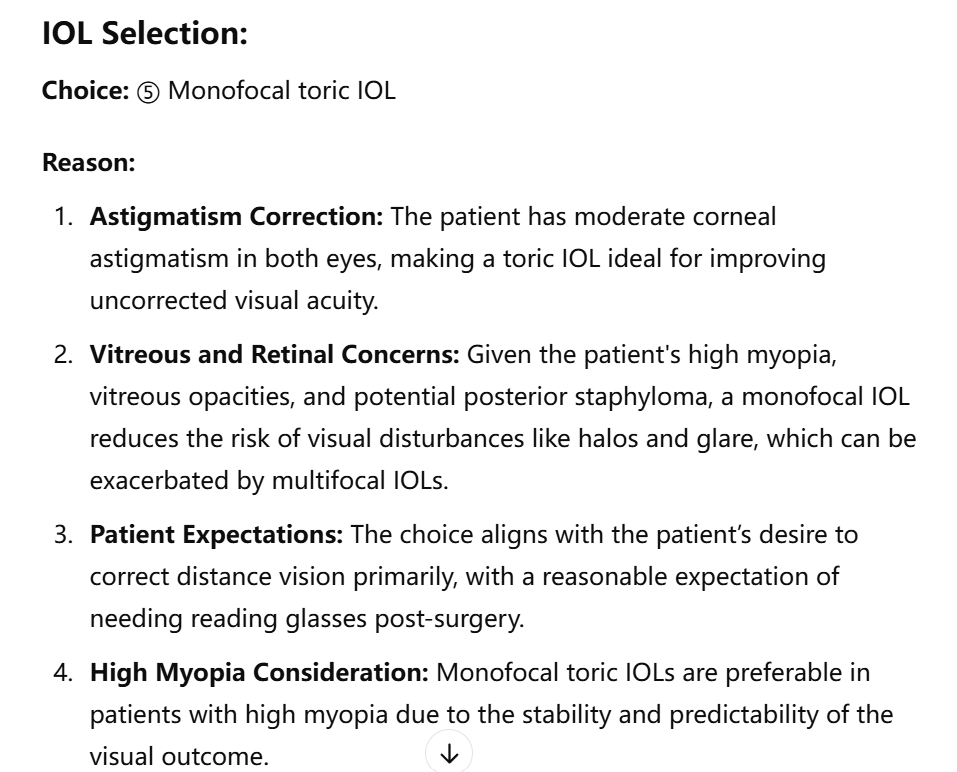

Supplement: Supplementary file 1 [file Data_Sheet_1.zip › supplementary material 3/89/σ▒Åσ╣òμê¬σ¢╛ 2024-08-09 063721.png]

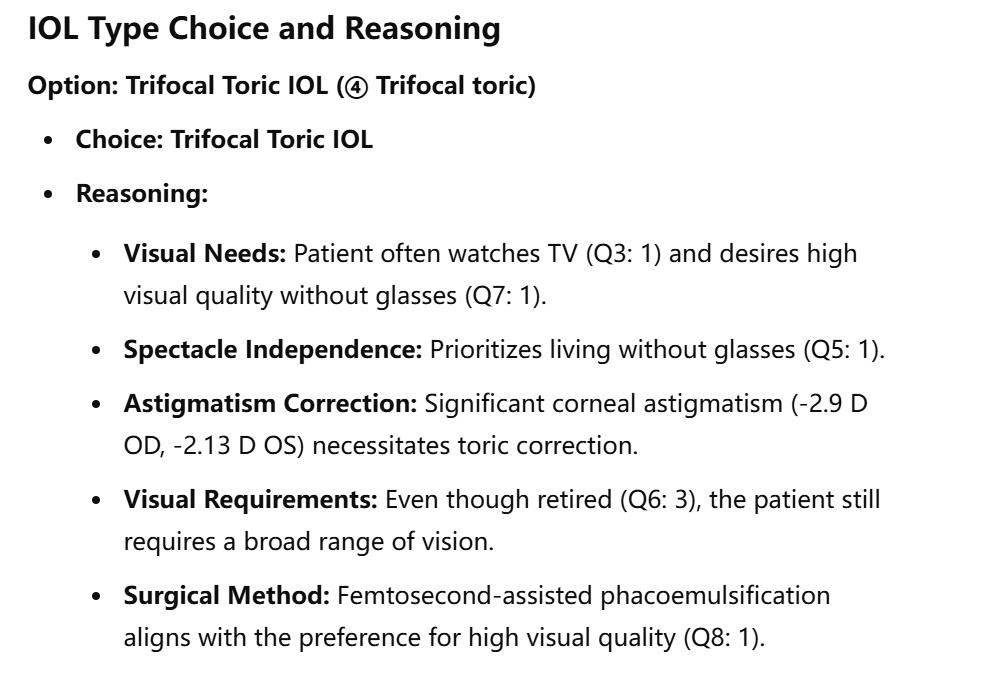

Supplement: Supplementary file 1 [file Data_Sheet_1.zip › supplementary material 3/116/σ▒Åσ╣òμê¬σ¢╛ 2024-08-05 153006.png]

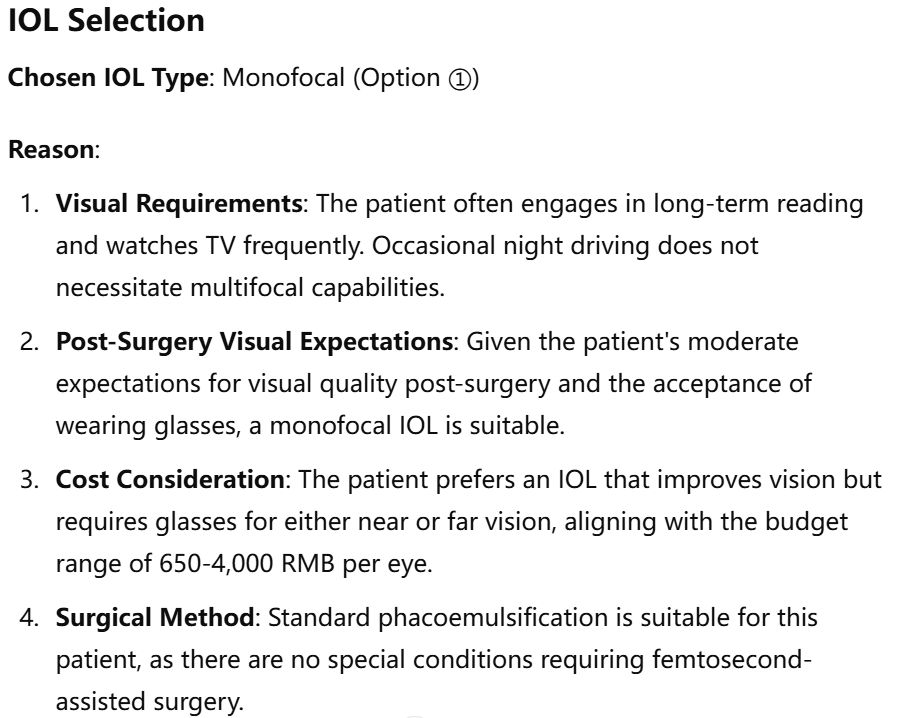

Supplement: Supplementary file 1 [file Data_Sheet_1.zip › supplementary material 3/118/σ▒Åσ╣òμê¬σ¢╛ 2024-08-05 153345.png]

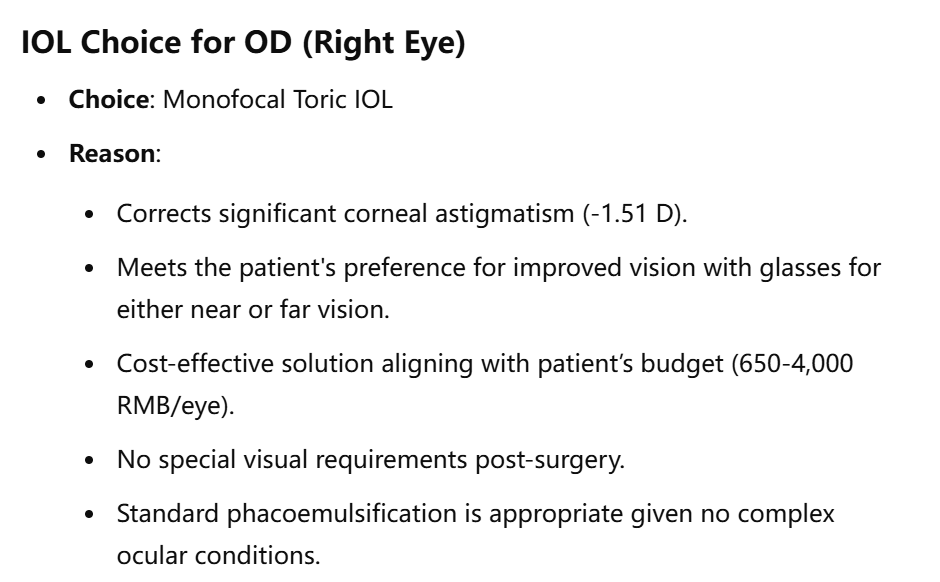

Supplement: Supplementary file 1 [file Data_Sheet_1.zip › supplementary material 3/120/σ▒Åσ╣òμê¬σ¢╛ 2024-08-05 153752.png]

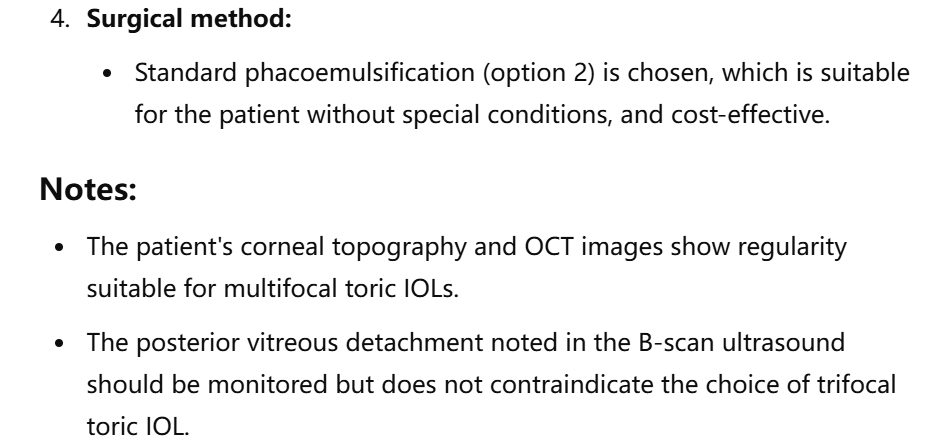

Supplement: Supplementary file 1 [file Data_Sheet_1.zip › supplementary material 3/144/σ▒Åσ╣òμê¬σ¢╛ 2024-08-06 023551.png]

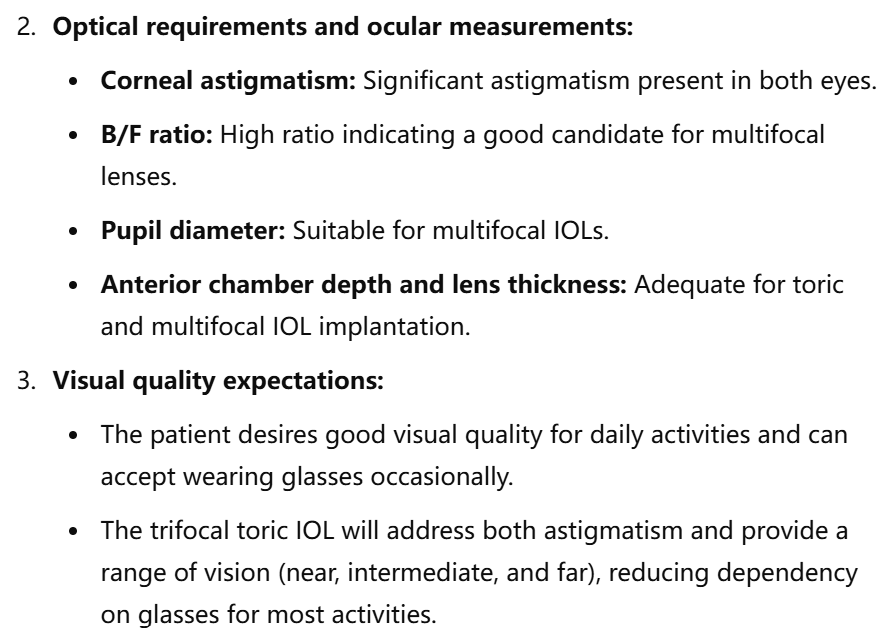

Supplement: Supplementary file 1 [file Data_Sheet_1.zip › supplementary material 3/144/σ▒Åσ╣òμê¬σ¢╛ 2024-08-06 023538.png]

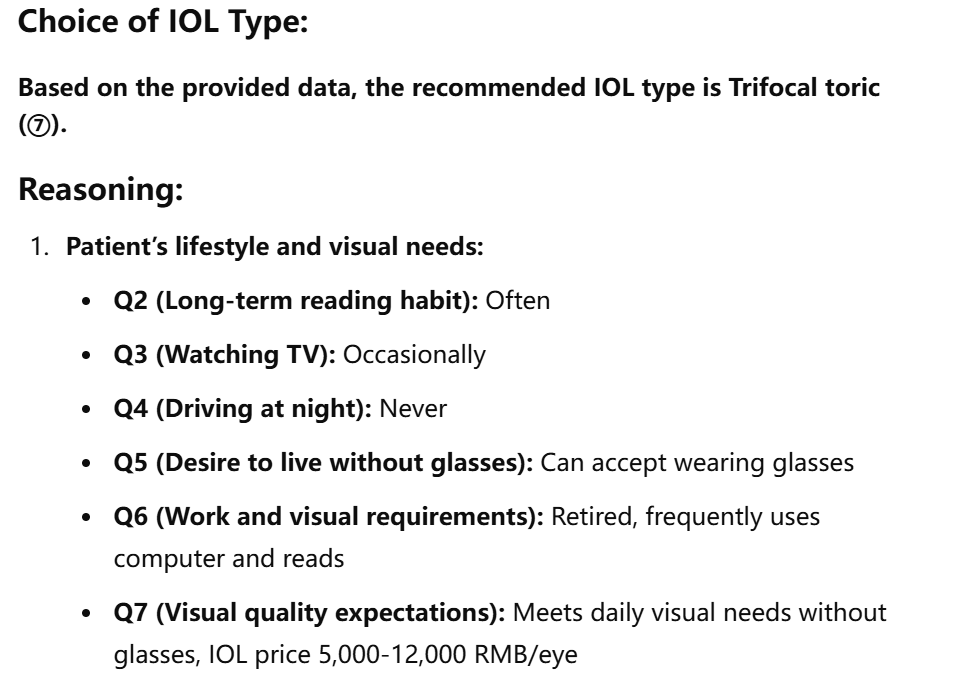

Supplement: Supplementary file 1 [file Data_Sheet_1.zip › supplementary material 3/144/σ▒Åσ╣òμê¬σ¢╛ 2024-08-06 023516.png]

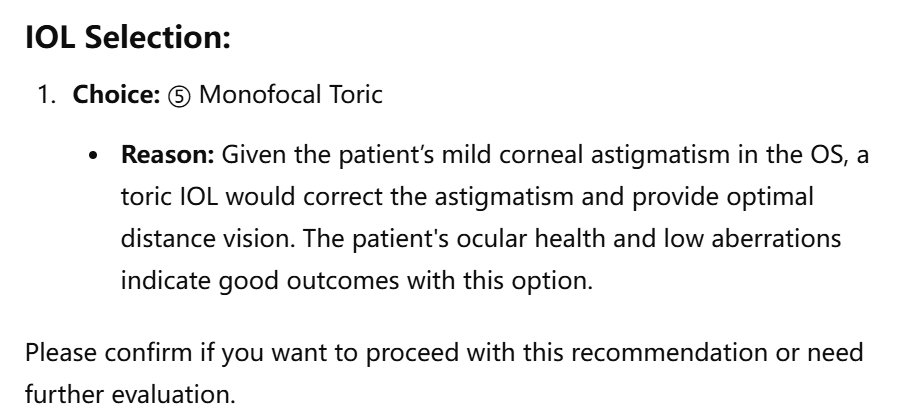

Supplement: Supplementary file 1 [file Data_Sheet_1.zip › supplementary material 3/21/σ▒Åσ╣òμê¬σ¢╛ 2024-08-09 063100.png]

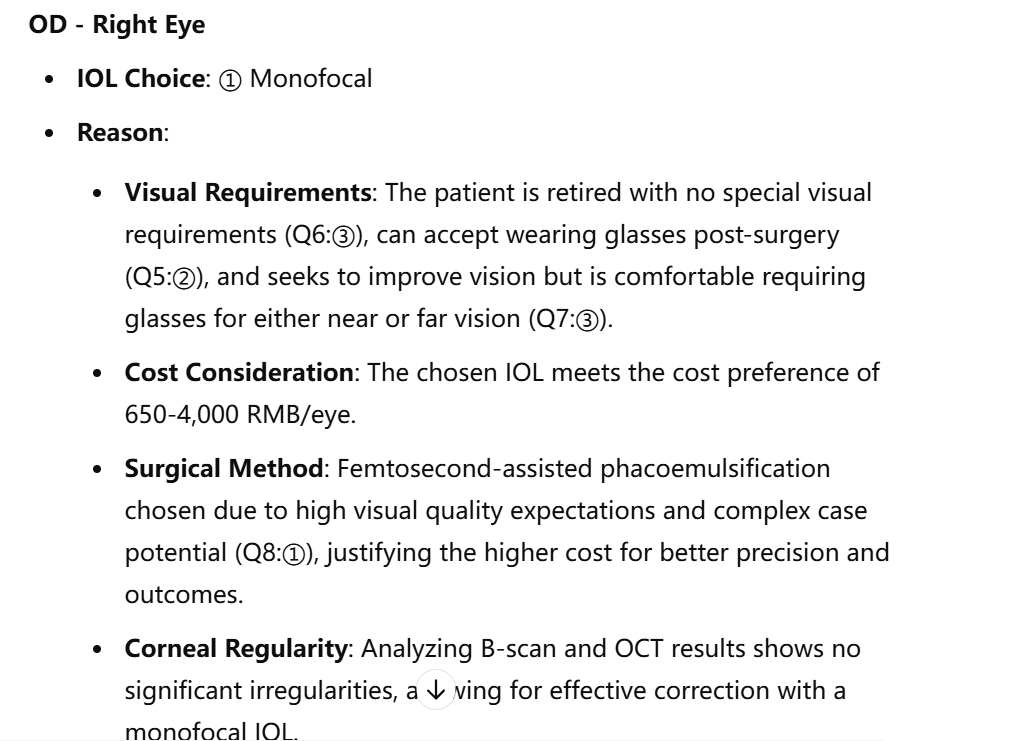

Supplement: Supplementary file 1 [file Data_Sheet_1.zip › supplementary material 3/126/σ▒Åσ╣òμê¬σ¢╛ 2024-08-06 005005.png]

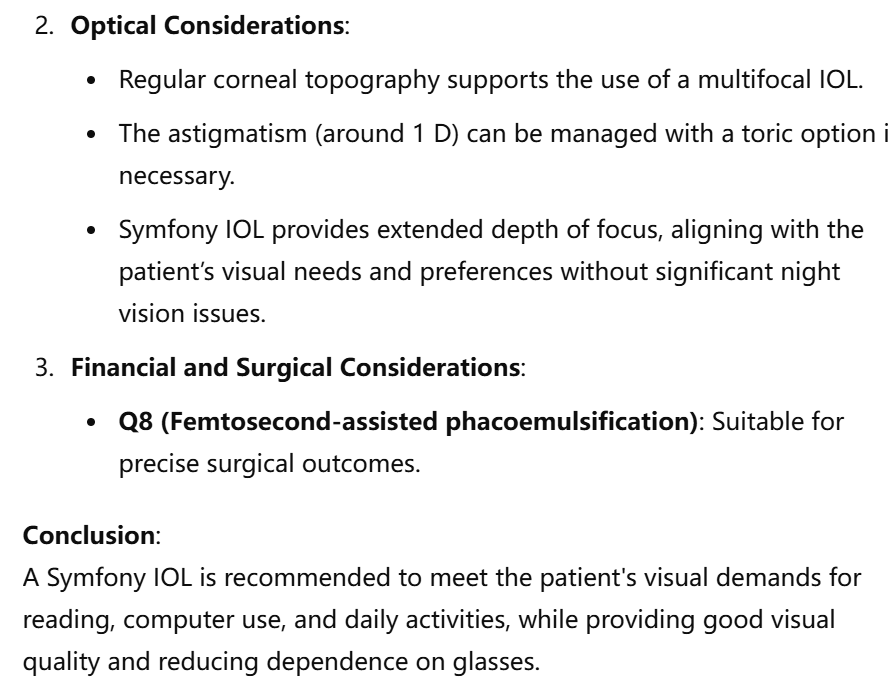

Supplement: Supplementary file 1 [file Data_Sheet_1.zip › supplementary material 3/110/1722756418348.jpg]

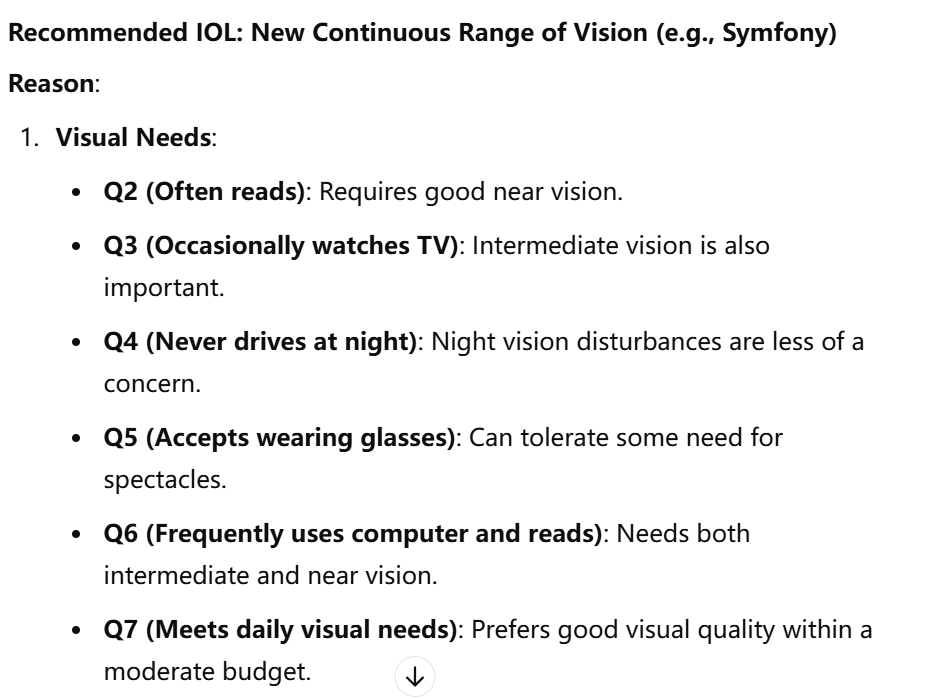

Supplement: Supplementary file 1 [file Data_Sheet_1.zip › supplementary material 3/110/1722756370678.jpg]

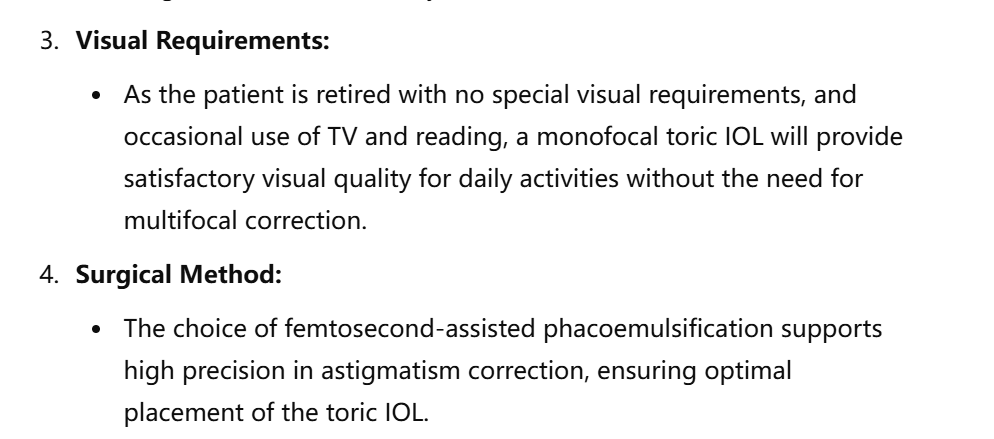

Supplement: Supplementary file 1 [file Data_Sheet_1.zip › supplementary material 3/128/σ▒Åσ╣òμê¬σ¢╛ 2024-08-06 015527.png]

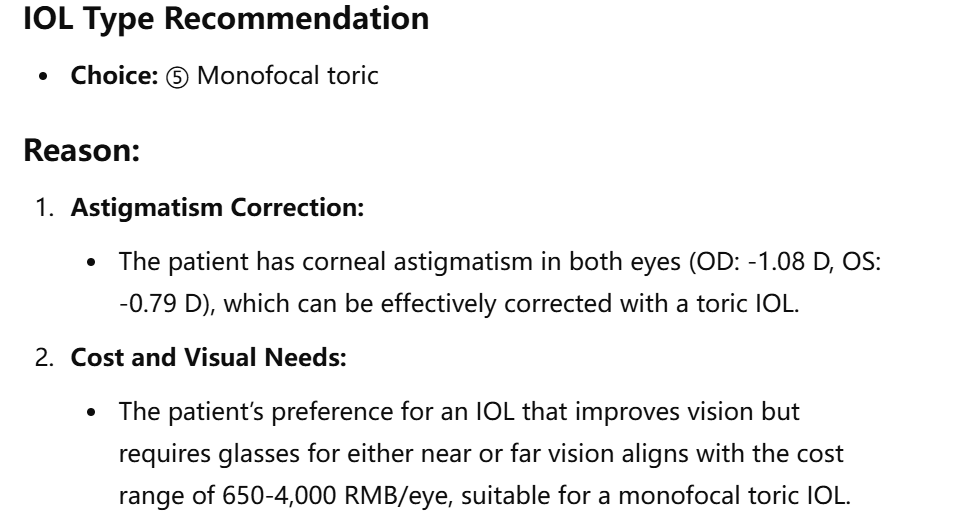

Supplement: Supplementary file 1 [file Data_Sheet_1.zip › supplementary material 3/128/σ▒Åσ╣òμê¬σ¢╛ 2024-08-06 015514.png]

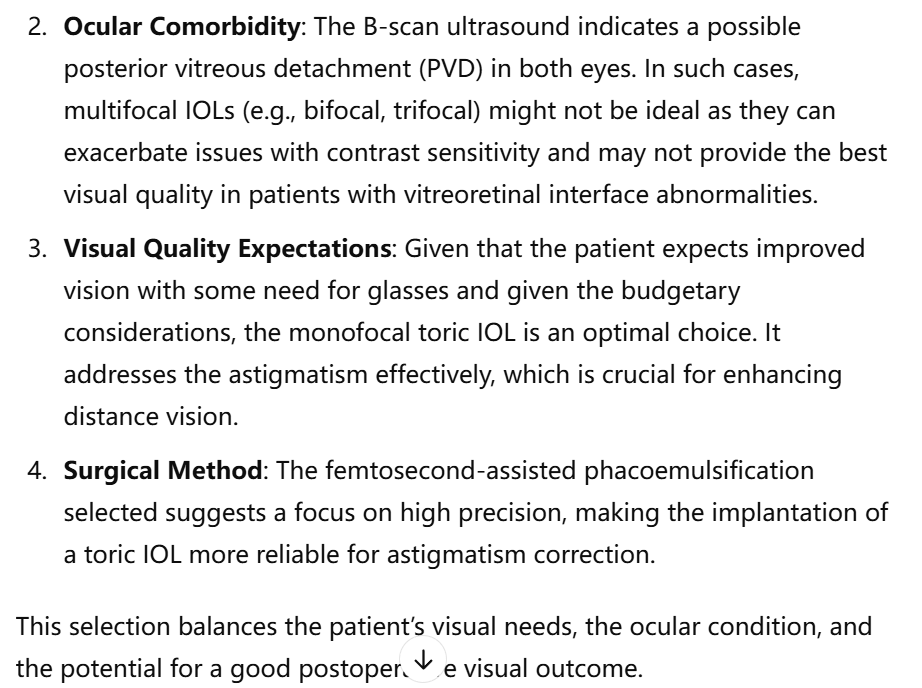

Supplement: Supplementary file 1 [file Data_Sheet_1.zip › supplementary material 3/91/σ▒Åσ╣òμê¬σ¢╛ 2024-08-09 064038.png]

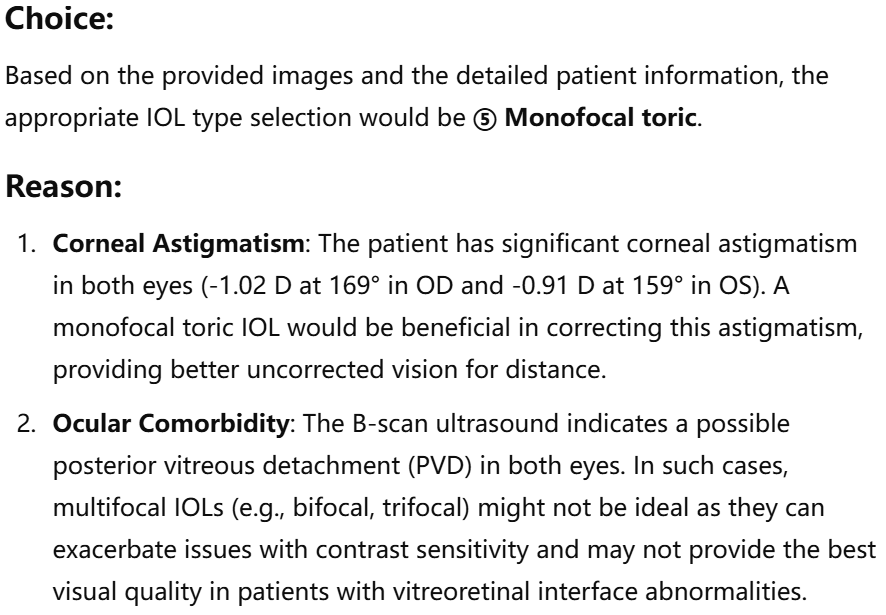

Supplement: Supplementary file 1 [file Data_Sheet_1.zip › supplementary material 3/91/σ▒Åσ╣òμê¬σ¢╛ 2024-08-09 064013.png]

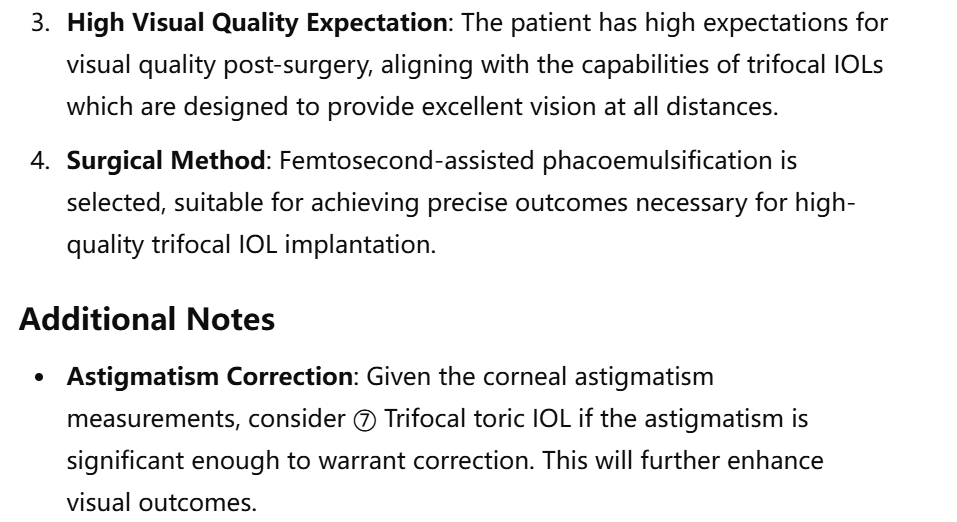

Supplement: Supplementary file 1 [file Data_Sheet_1.zip › supplementary material 3/136/σ▒Åσ╣òμê¬σ¢╛ 2024-08-06 022104.png]

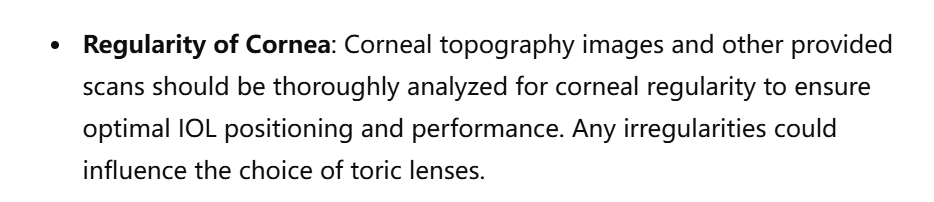

Supplement: Supplementary file 1 [file Data_Sheet_1.zip › supplementary material 3/136/σ▒Åσ╣òμê¬σ¢╛ 2024-08-06 022121.png]

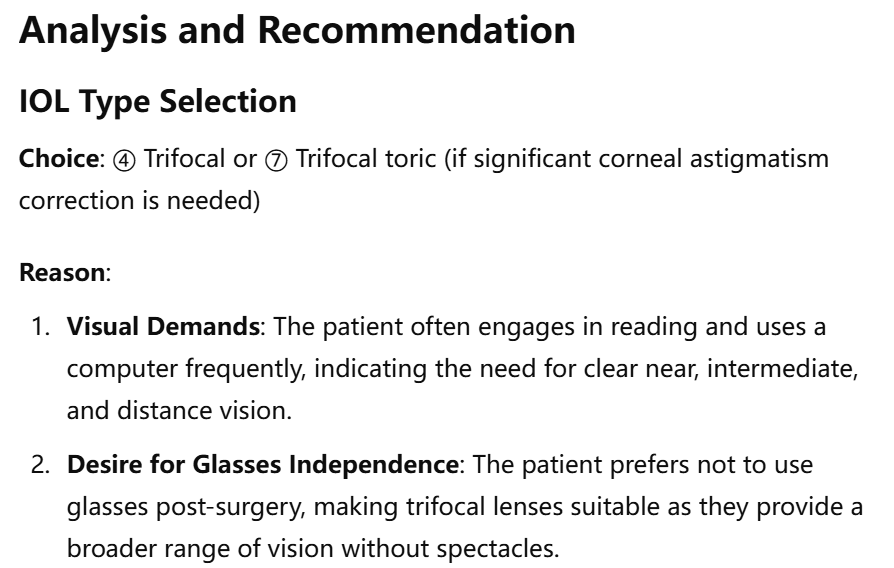

Supplement: Supplementary file 1 [file Data_Sheet_1.zip › supplementary material 3/136/σ▒Åσ╣òμê¬σ¢╛ 2024-08-06 022042.png]

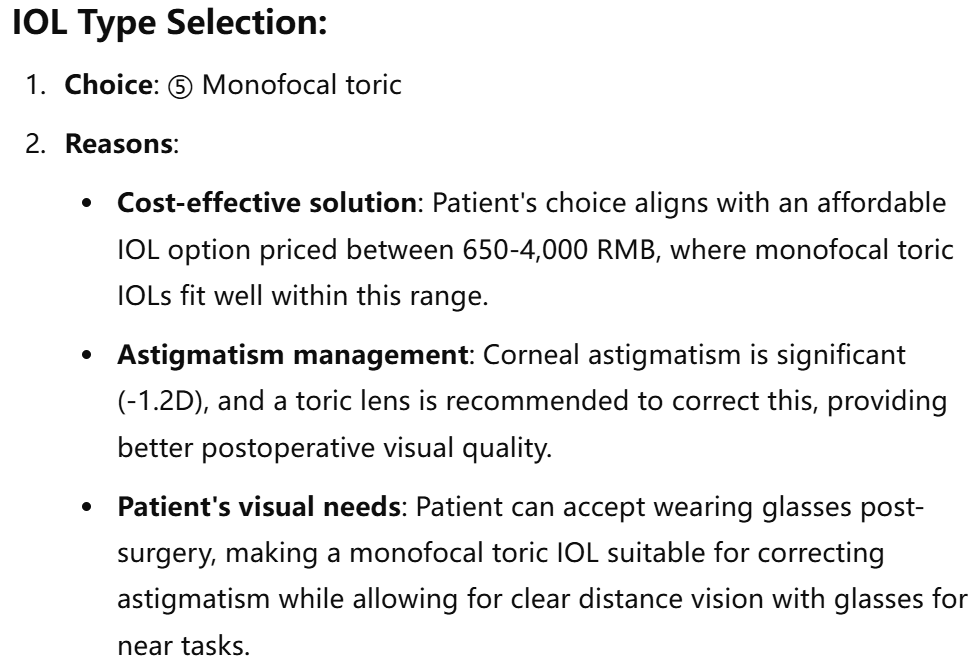

Supplement: Supplementary file 1 [file Data_Sheet_1.zip › supplementary material 3/100/σ▒Åσ╣òμê¬σ¢╛ 2024-08-09 065510.png]

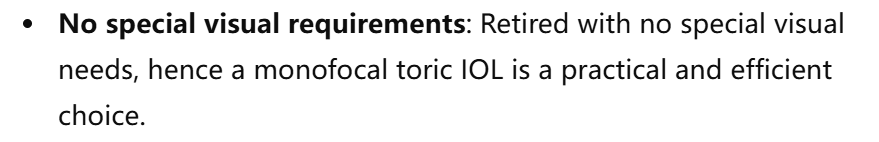

Supplement: Supplementary file 1 [file Data_Sheet_1.zip › supplementary material 3/100/σ▒Åσ╣òμê¬σ¢╛ 2024-08-09 065528.png]

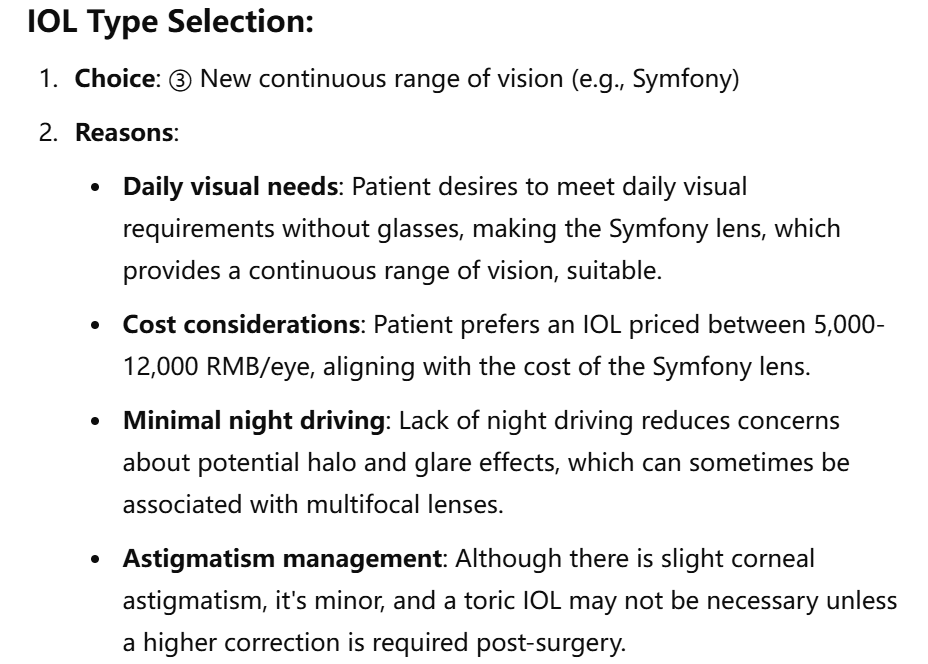

Supplement: Supplementary file 1 [file Data_Sheet_1.zip › supplementary material 3/98/σ▒Åσ╣òμê¬σ¢╛ 2024-08-09 065221.png]

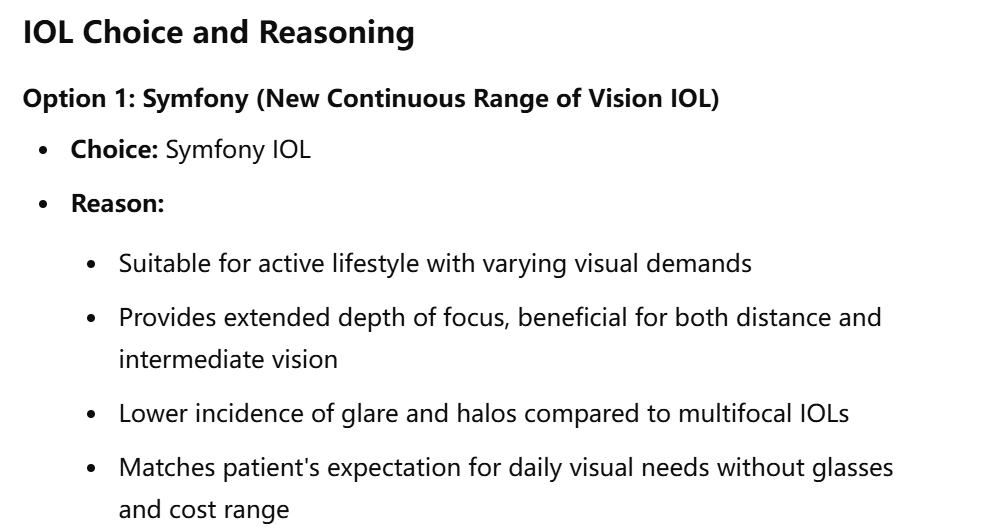

Supplement: Supplementary file 1 [file Data_Sheet_1.zip › supplementary material 3/138/σ▒Åσ╣òμê¬σ¢╛ 2024-08-06 022358.png]

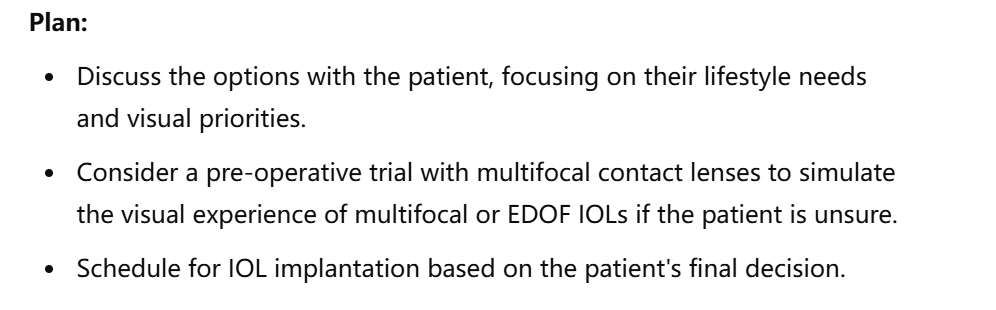

Supplement: Supplementary file 1 [file Data_Sheet_1.zip › supplementary material 3/138/σ▒Åσ╣òμê¬σ¢╛ 2024-08-06 022501.png]

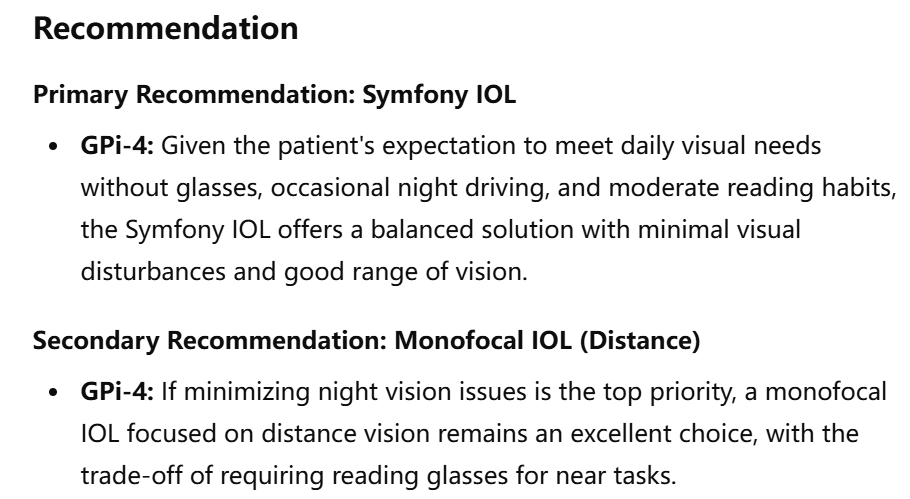

Supplement: Supplementary file 1 [file Data_Sheet_1.zip › supplementary material 3/138/σ▒Åσ╣òμê¬σ¢╛ 2024-08-06 022452.png]

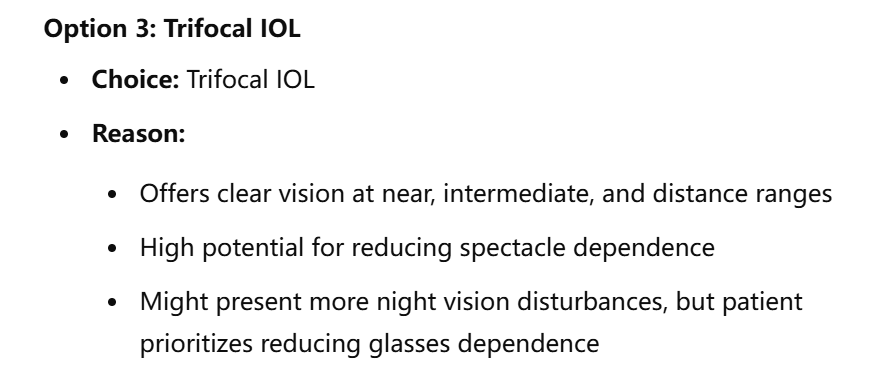

Supplement: Supplementary file 1 [file Data_Sheet_1.zip › supplementary material 3/138/σ▒Åσ╣òμê¬σ¢╛ 2024-08-06 022430.png]

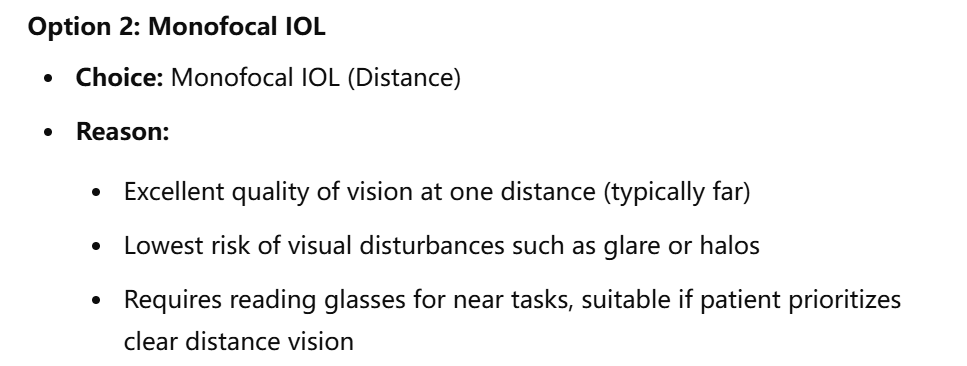

Supplement: Supplementary file 1 [file Data_Sheet_1.zip › supplementary material 3/138/σ▒Åσ╣òμê¬σ¢╛ 2024-08-06 022418.png]

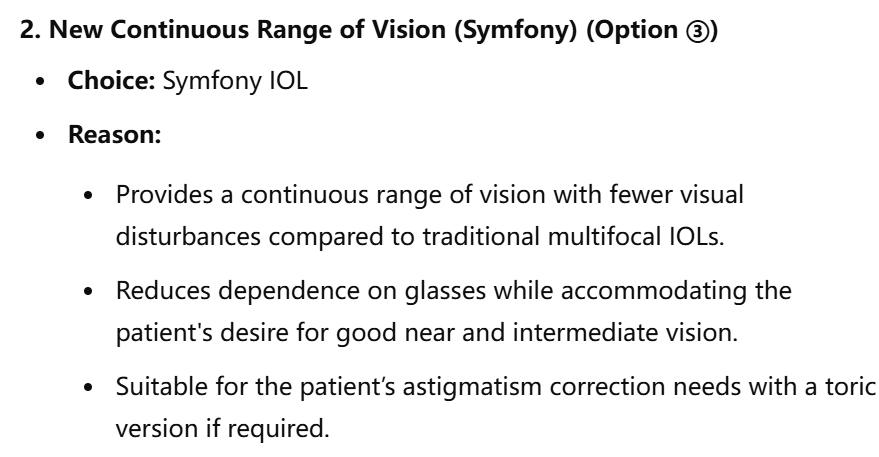

Supplement: Supplementary file 1 [file Data_Sheet_1.zip › supplementary material 3/106/1722755557349.jpg]

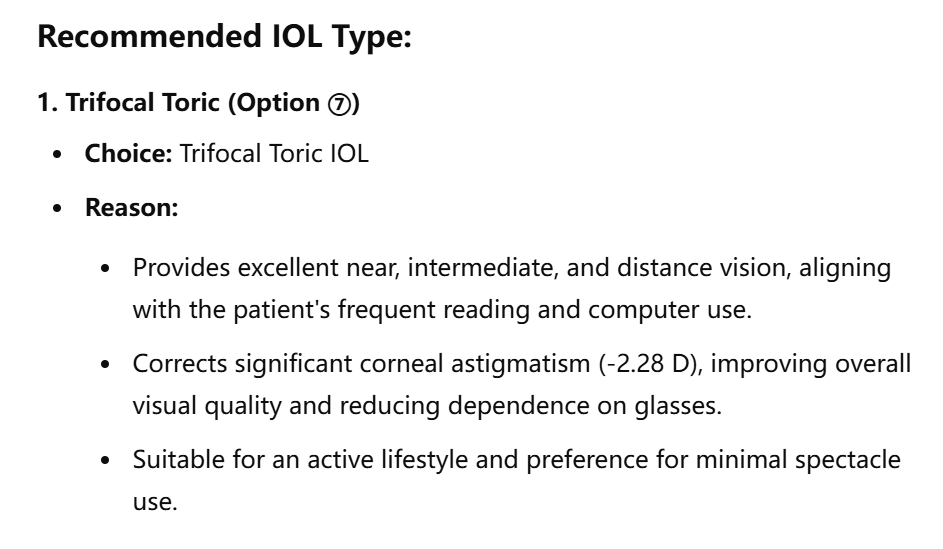

Supplement: Supplementary file 1 [file Data_Sheet_1.zip › supplementary material 3/106/4f9b5783414aa2f6eb1ce55600c81d2.png]

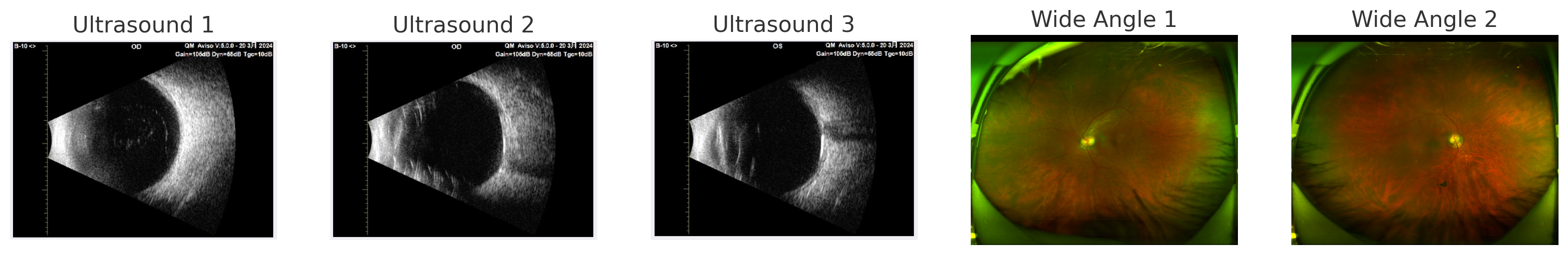

Supplement: Supplementary file 1 [file Data_Sheet_1.zip › supplementary material 3/106/output.png]

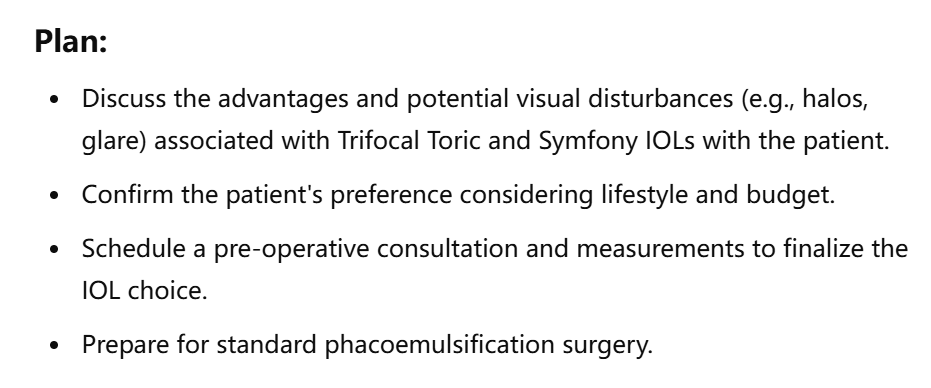

Supplement: Supplementary file 1 [file Data_Sheet_1.zip › supplementary material 3/106/1722755723719.jpg]

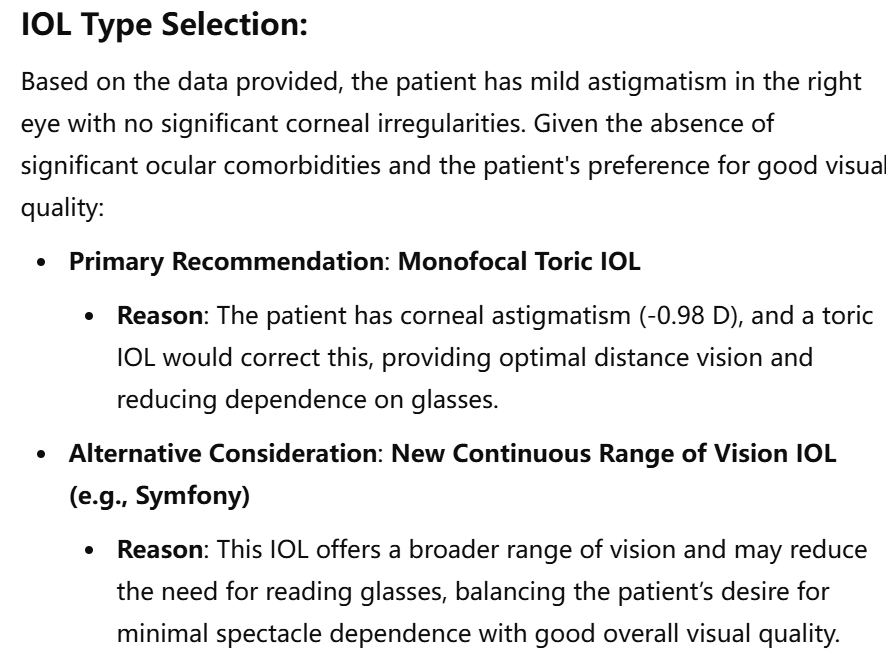

Supplement: Supplementary file 1 [file Data_Sheet_1.zip › supplementary material 3/97/σ▒Åσ╣òμê¬σ¢╛ 2024-08-09 064952.png]

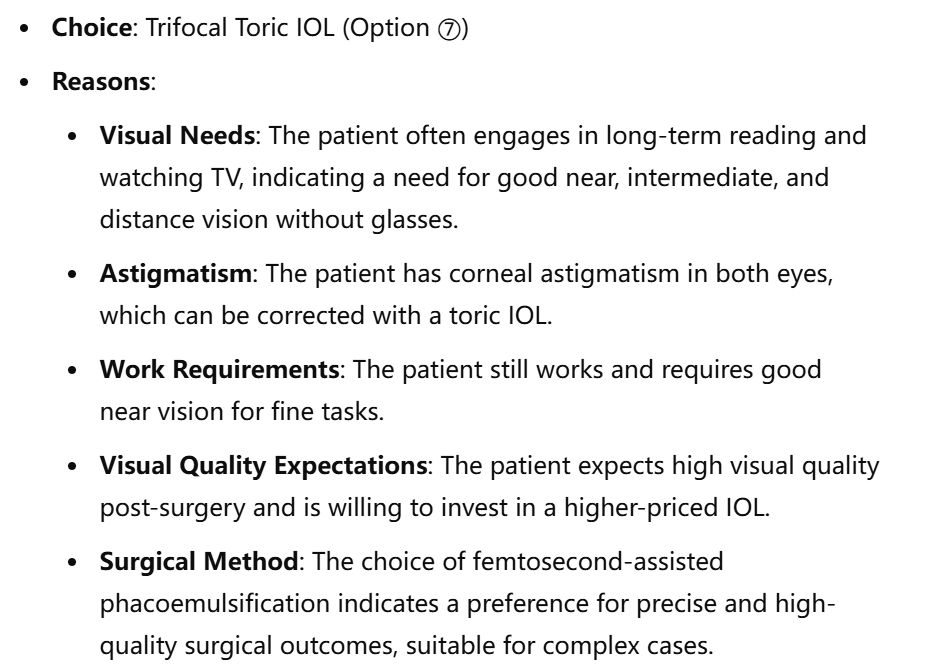

Supplement: Supplementary file 1 [file Data_Sheet_1.zip › supplementary material 3/108/1722755975515.jpg]

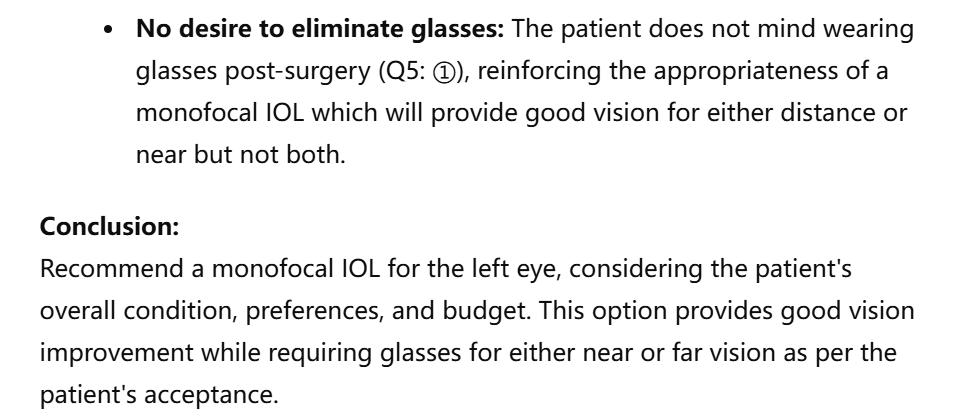

Supplement: Supplementary file 1 [file Data_Sheet_1.zip › supplementary material 3/130/σ▒Åσ╣òμê¬σ¢╛ 2024-08-06 015900.png]

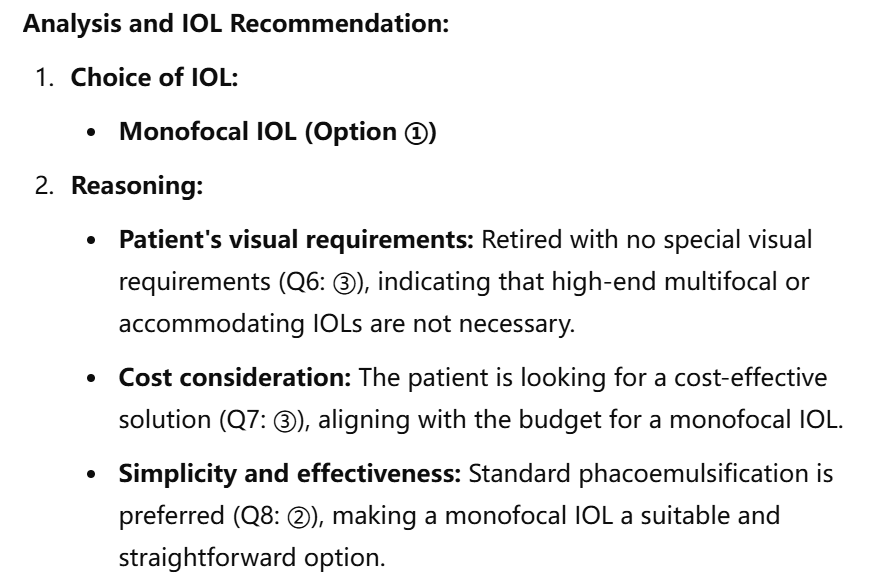

Supplement: Supplementary file 1 [file Data_Sheet_1.zip › supplementary material 3/130/σ▒Åσ╣òμê¬σ¢╛ 2024-08-06 015822.png]

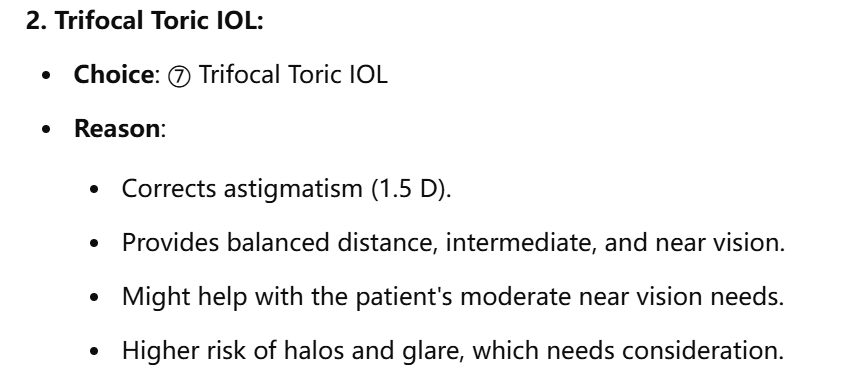

Supplement: Supplementary file 1 [file Data_Sheet_1.zip › supplementary material 3/112/1722756898353.jpg]

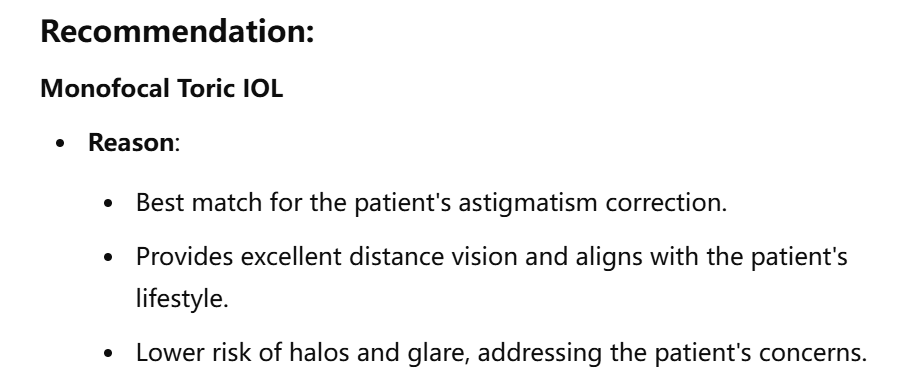

Supplement: Supplementary file 1 [file Data_Sheet_1.zip › supplementary material 3/112/1722756934508.jpg]

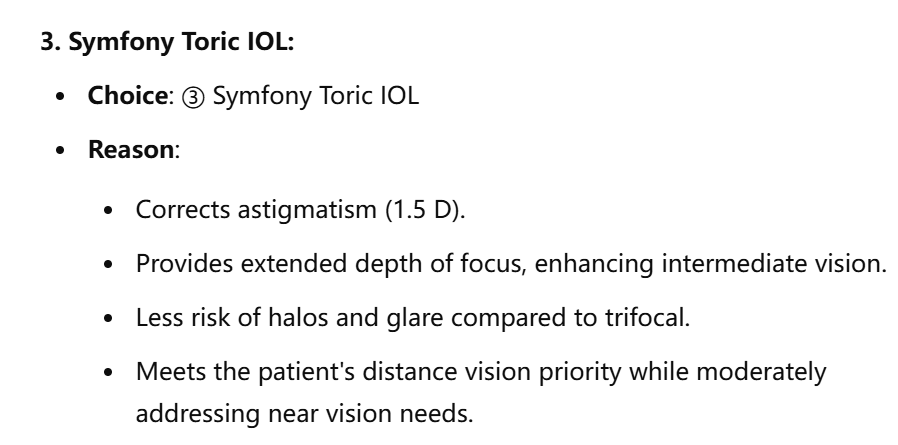

Supplement: Supplementary file 1 [file Data_Sheet_1.zip › supplementary material 3/112/1722756916457.jpg]

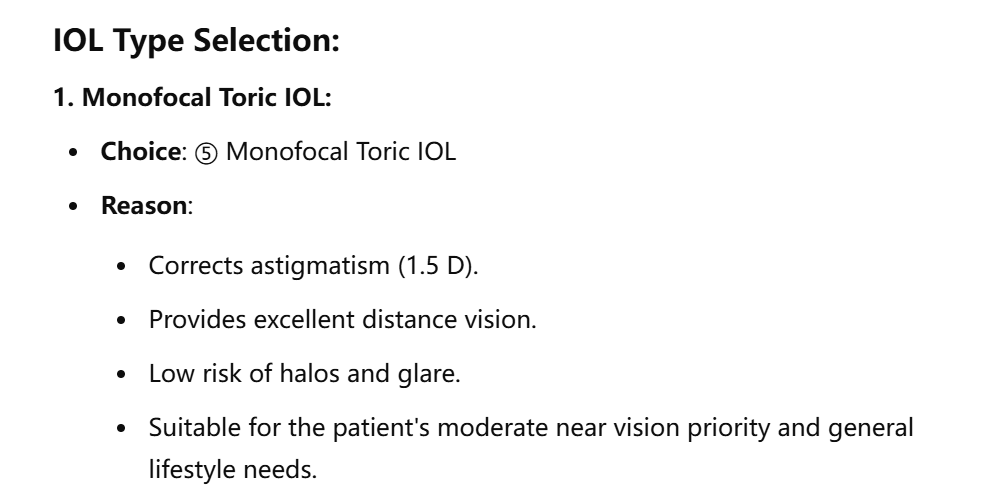

Supplement: Supplementary file 1 [file Data_Sheet_1.zip › supplementary material 3/112/1722756863372.jpg]

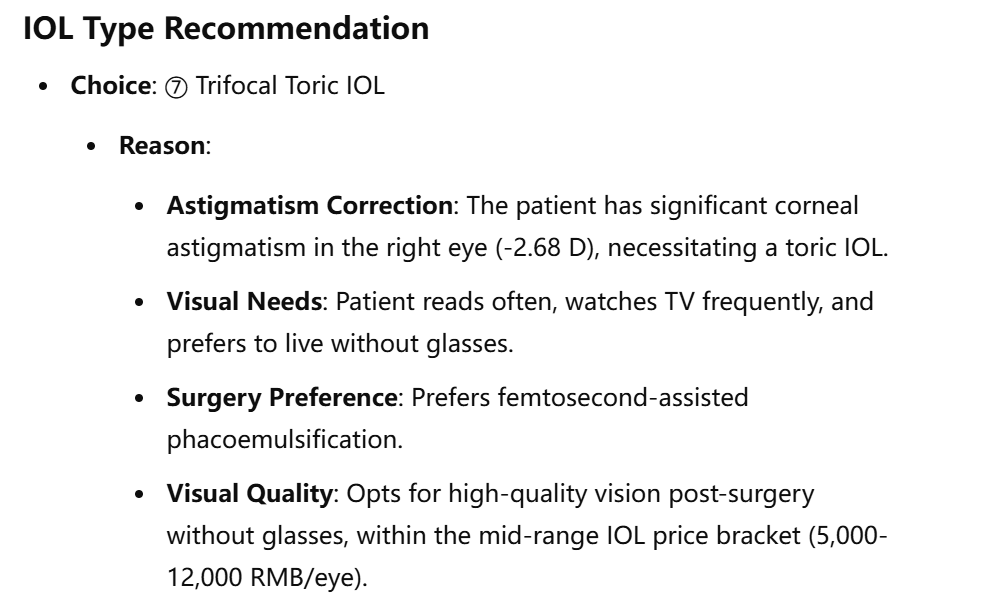

Supplement: Supplementary file 1 [file Data_Sheet_1.zip › supplementary material 3/124/σ▒Åσ╣òμê¬σ¢╛ 2024-08-05 154520.png]

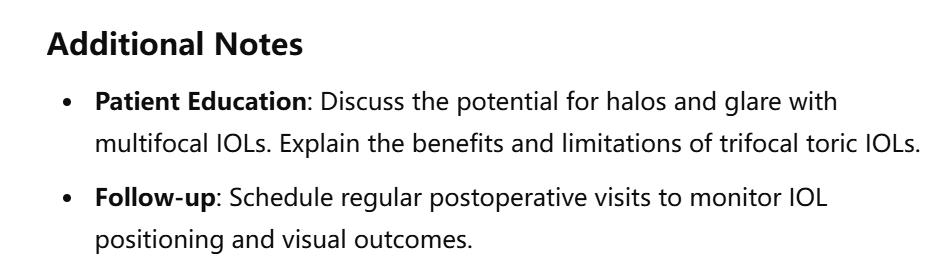

Supplement: Supplementary file 1 [file Data_Sheet_1.zip › supplementary material 3/124/σ▒Åσ╣òμê¬σ¢╛ 2024-08-05 154533.png]

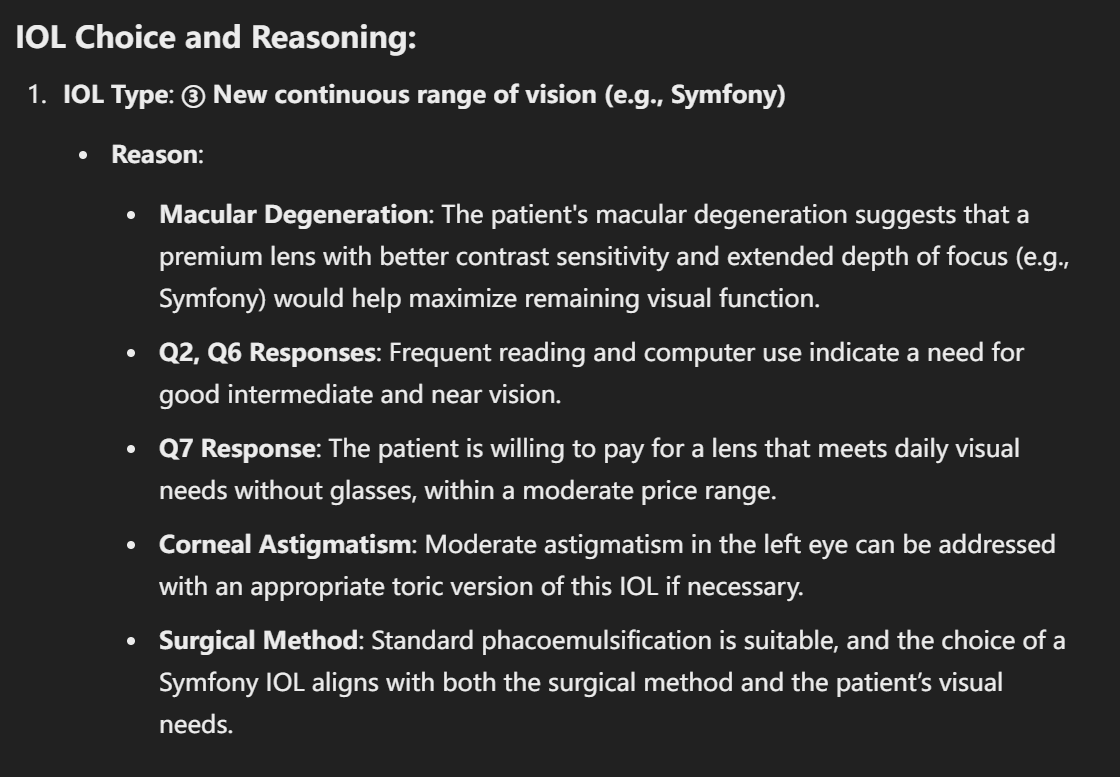

Supplement: Supplementary file 1 [file Data_Sheet_1.zip › supplementary material 3/23/4.png]

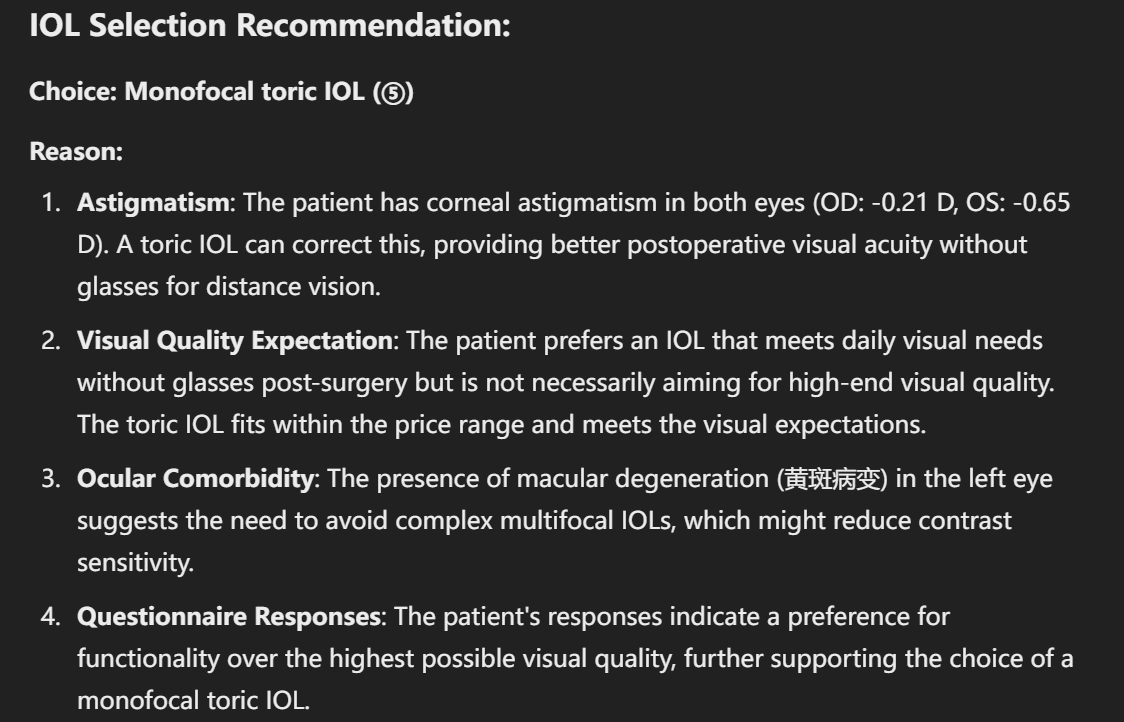

Supplement: Supplementary file 1 [file Data_Sheet_1.zip › supplementary material 3/23/5.png]

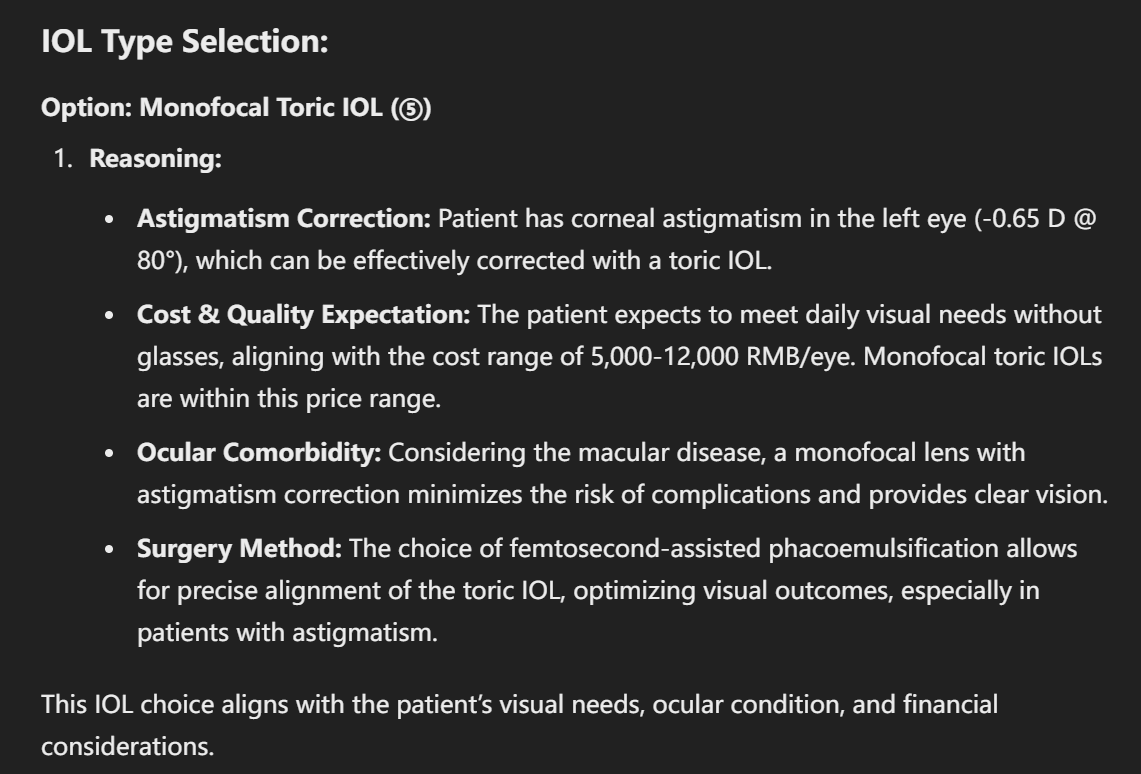

Supplement: Supplementary file 1 [file Data_Sheet_1.zip › supplementary material 3/23/2.png]

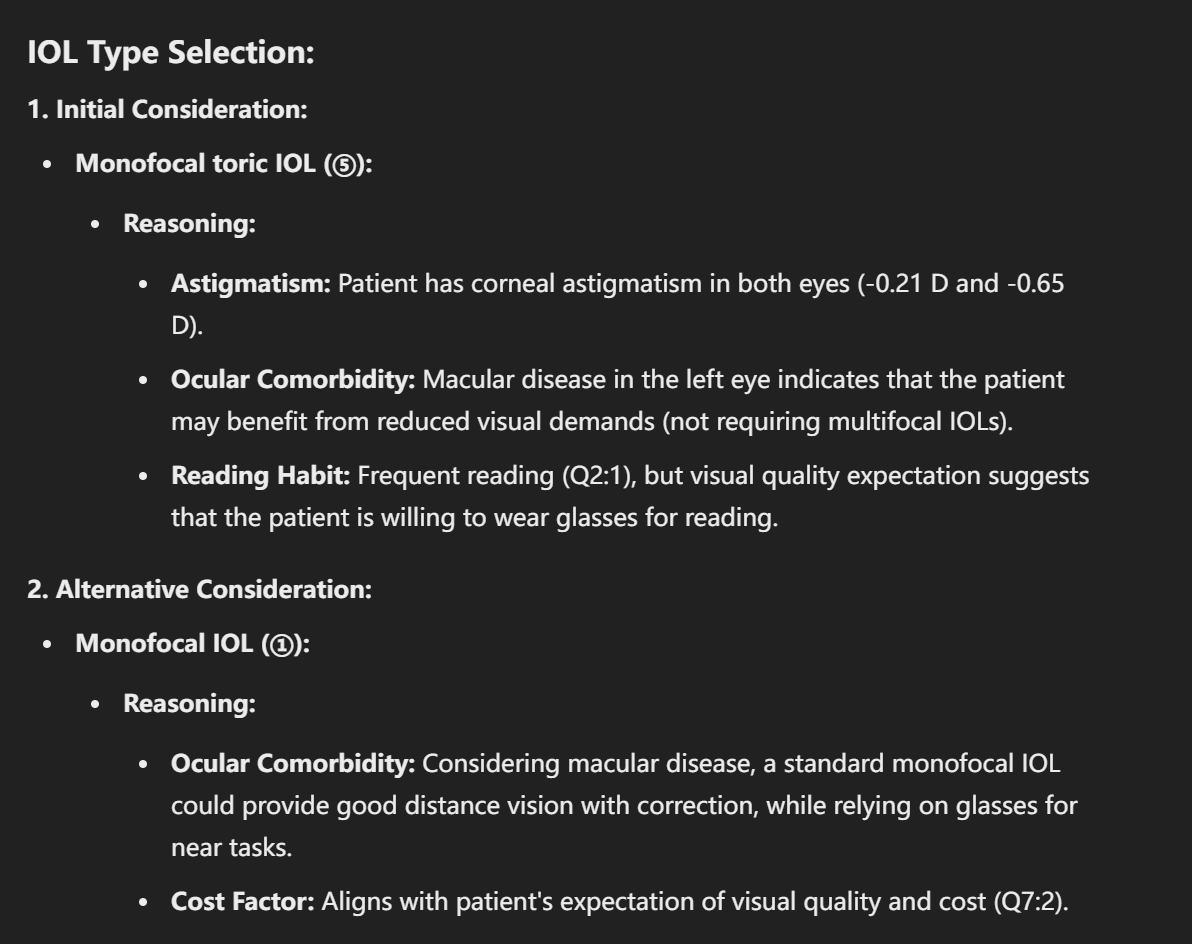

Supplement: Supplementary file 1 [file Data_Sheet_1.zip › supplementary material 3/23/3.png]

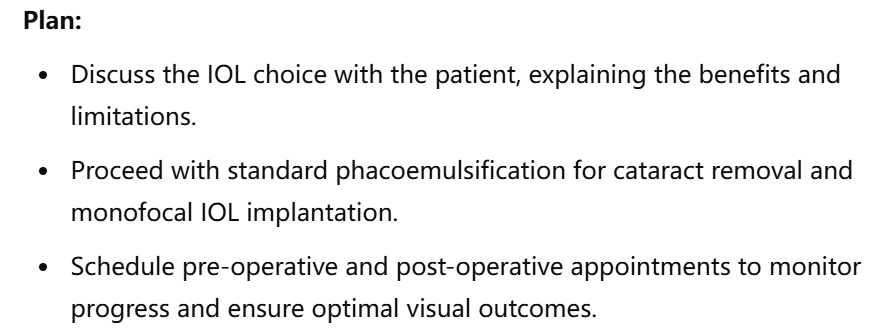

Supplement: Supplementary file 1 [file Data_Sheet_1.zip › supplementary material 3/146/σ▒Åσ╣òμê¬σ¢╛ 2024-08-06 024049.png]

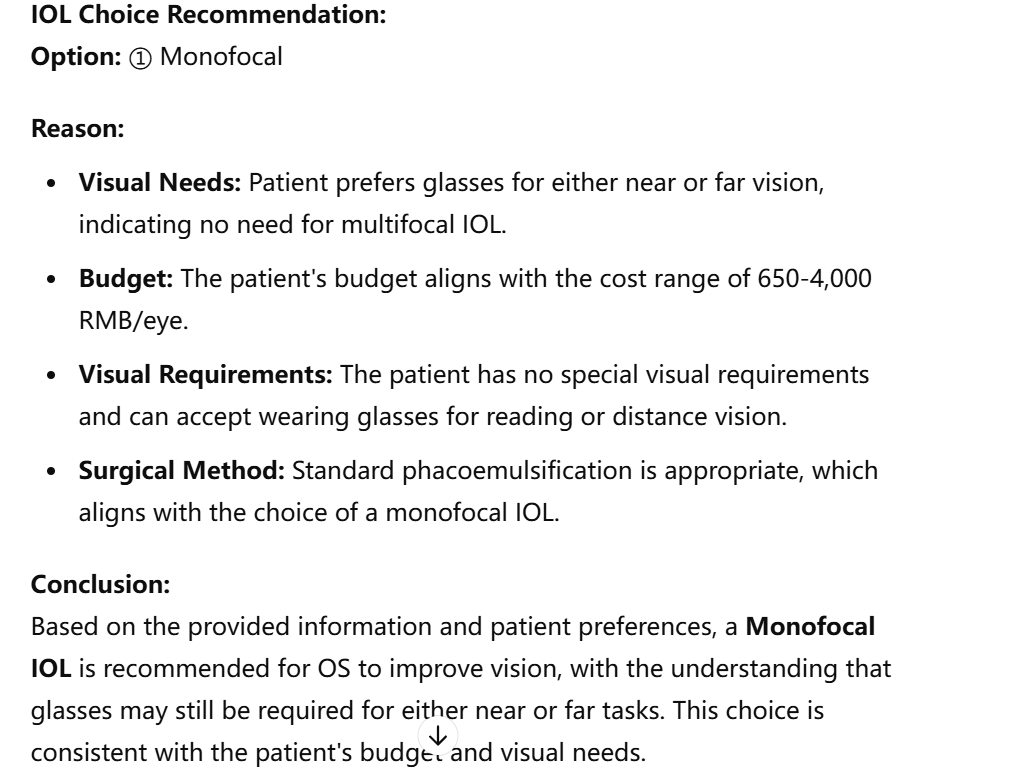

Supplement: Supplementary file 1 [file Data_Sheet_1.zip › supplementary material 3/146/σ▒Åσ╣òμê¬σ¢╛ 2024-08-06 024031.png]

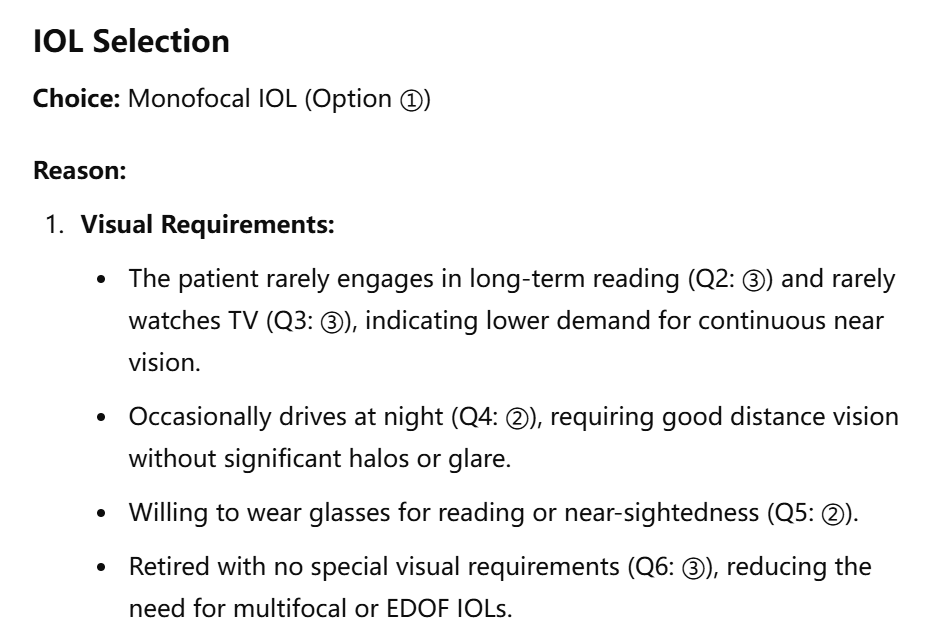

Supplement: Supplementary file 1 [file Data_Sheet_1.zip › supplementary material 3/122/σ▒Åσ╣òμê¬σ¢╛ 2024-08-05 154114.png]

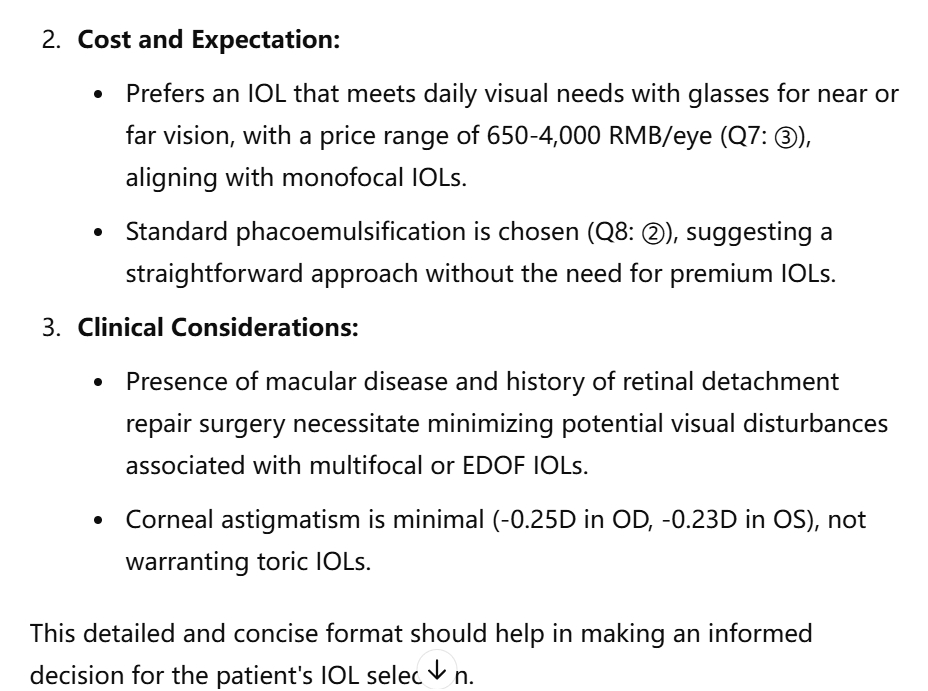

Supplement: Supplementary file 1 [file Data_Sheet_1.zip › supplementary material 3/122/σ▒Åσ╣òμê¬σ¢╛ 2024-08-05 154134.png]

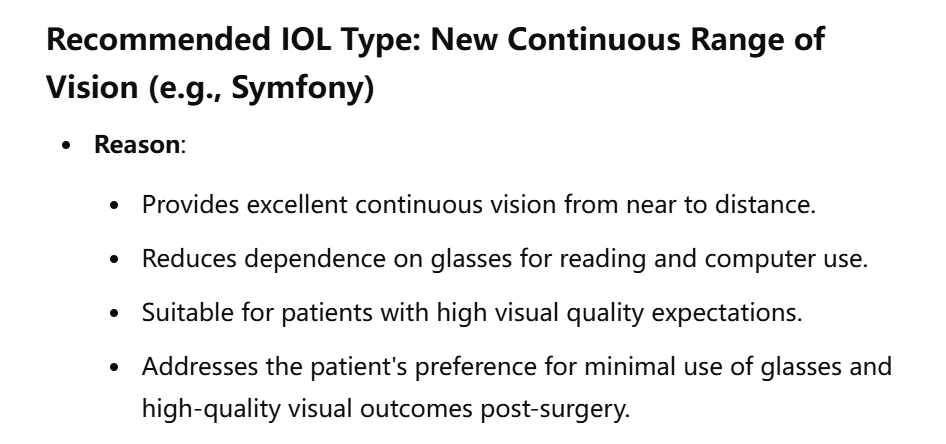

Supplement: Supplementary file 1 [file Data_Sheet_1.zip › supplementary material 3/114/1722757174451.jpg]

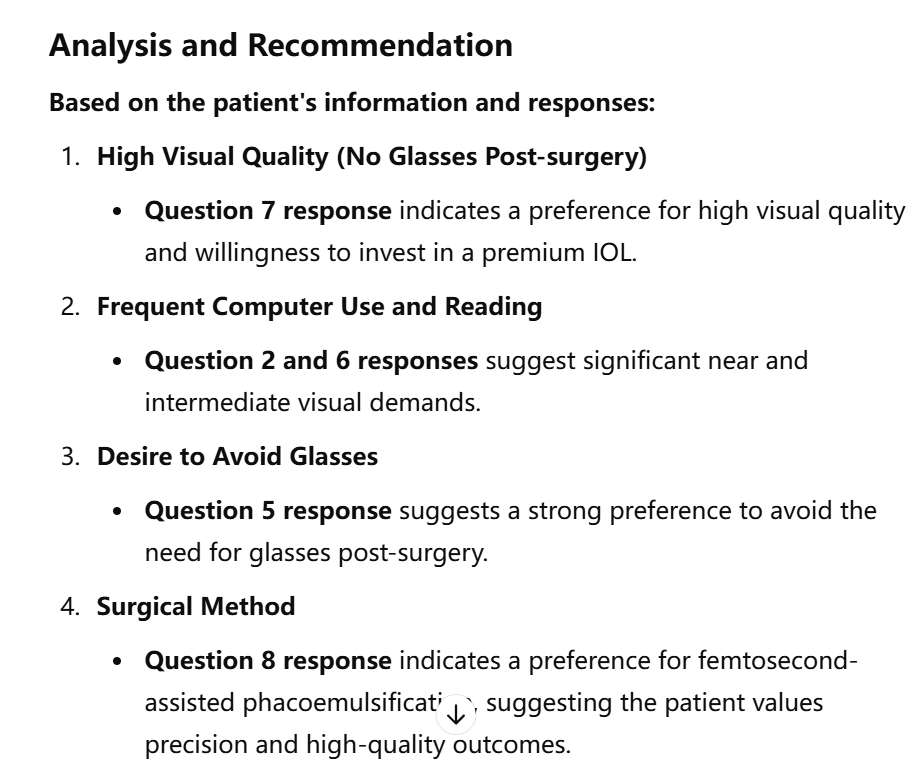

Supplement: Supplementary file 1 [file Data_Sheet_1.zip › supplementary material 3/114/1722757127142.jpg]

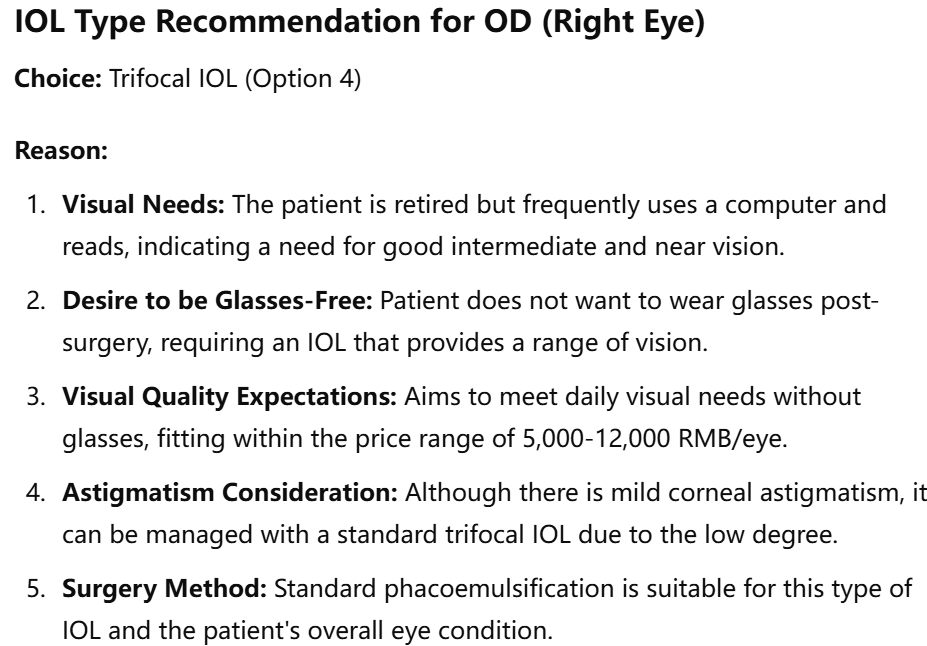

Supplement: Supplementary file 1 [file Data_Sheet_1.zip › supplementary material 3/140/σ▒Åσ╣òμê¬σ¢╛ 2024-08-06 022802.png]

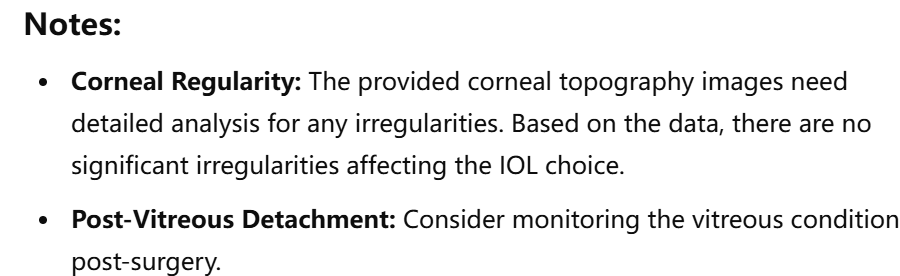

Supplement: Supplementary file 1 [file Data_Sheet_1.zip › supplementary material 3/140/σ▒Åσ╣òμê¬σ¢╛ 2024-08-06 022813.png]
