# Supplementary material for: Assessing GPT-4o in cataract surgery decision-making: appropriateness, consistency, and clinical implications
Source: Front Artif Intell. 2026 May 29;9:1810899. doi: 10.3389/frai.2026.1810899 (PMC13260408; doi:10.3389/frai.2026.1810899)
Supplement: Supplementary file 2 [file Data_Sheet_2.docx]

**GPT-4o Prompt**

**Question 1:** I am an ophthalmologist. You are assisting me in making medical choices. Answer questions based on the provided information. Ensure answers are succinct, accurate, and consistent. Use EBM（Evidence-based medicine）where appropriate（like guides from AAO and ESCRS）. Separate the choice and reason into main points. Refer to all respondents with abbreviations like GPi-4 and real ophthalmologists to avoid discrimination. Do you understand?

Provide options and reasons step by step according to the patient's basic information and questionnaire responses. Be specific and emphasize brevity. Organize responses as an ophthalmologist's hospital notes, focusing on the choice of IOL type. Use abbreviations and keep the format concise. Abbreviations do not need to be defined.

**Question 2:** I will provide all questions and options initially. Subsequently, I will provide only the serial number of the options. Do you understand?" Questions and Options: Question 2: Do you have a habit of long-term reading (books, newspapers, mobile phones)? ① Often ② Occasionally ③ Never Question 3: Do you watch TV frequently? ① Often ② Occasionally ③ Never Question 4: Do you drive at night? ① Often ② Occasionally ③ Never Question 5: Do you want to live without glasses (no reading glasses/near-sighted glasses)? ① No ② Can accept wearing reading or near-sighted glasses Question 6: Working condition? ① Still working, requires close and fine work ② Retired, but frequently uses computer and reads ③ Retired, no special visual requirements Question 7: Visual quality expectations for implanted IOL after cataract removal ① Expect high visual quality (no glasses post-surgery), IOL price around 20,000 RMB/eye ② Meets daily visual needs (no glasses post-surgery), IOL price 5,000-12,000 RMB/eye ③ Improves vision, but requires glasses for either near or far vision, IOL price 650-4,000 RMB/eye Question 8: Cataract removal surgery method ① Femtosecond-assisted phacoemulsification (for complex cases, high visual quality expectations), cost 8,000 RMB ② Standard phacoemulsification (most patients without special conditions)

| **Post-operative lifestyle questionnaire for cataract patients** | | |
| --- | --- | --- |
| 1. **Age** 2. **Do you have a habit of long-term reading (books, newspapers, mobile phones)?** | | |
| ① Often | ② Occasionally | ③ Never |
| 1. **Do you watch TV frequently?** | | |
| ① Often | ② Occasionally | ③ Never |
| 1. **Do you drive at night?** | | |
| ① Often | ② Occasionally | ③ Never |
| 1. **Do you want to live without glasses (no reading glasses/near-sighted glasses)?** | | |
| 1. No | 1. Can accept wearing reading or near-sighted glasses |  |
| 1. **Working condition?** |  |  |
| 1. Still working, requires close and fine work | 1. Retired, but frequently uses computer and reads | 1. Retired, no special visual requirements |
| 1. **Visual quality expectations for implanted IOL after cataract removal** | | |
| 1. Expect high visual quality (no glasses post-surgery), IOL price around 20,000 RMB/eye | 1. Meets daily visual needs (no glasses post-surgery), IOL price 5,000-12,000 RMB/eye | 1. Improves vision, but requires glasses for either near or far vision, IOL price 650-4,000 RMB/eye |
| 1. **Cataract removal surgery method** | | |
| 1. Femtosecond-assisted phacoemulsification (for complex cases, high visual quality expectations), cost 8,000 RMB | 1. Standard phacoemulsification (most patients without special conditions) |  |

**Question 3:** I will provide basic patient information in the following format: Sex, Age, General condition, Eye for surgery, OD AL (mm), K flat (D), K flat axis (°),K steep (D), K steep axis (°), Corneal Astigmatism value(D), Corneal Astigmatism axis（°）, Anterior Chamber Depth(mm),Lens Thickness(mm),White to White(mm), κ angle(mm), α angle(mm), B/F ratio, pupil diameter（mm）, Corneal High-order Aberrations in 4mm diameter, Corneal Spherical aberration in 6 mm diameter , Uncorrected Visual Acuity, Best Corrected Visual Acuity , Ocular comorbidity, History of previous ocular surgery; OS AL (mm), K flat (D), K flat axis (°),K steep (D), K steep axis (°), Corneal Astigmatism value(D), Corneal Astigmatism axis（°）, Anterior Chamber Depth(mm),Lens Thickness(mm),White to White(mm), κ angle(mm), α angle(mm), B/F ratio, pupil diameter（mm）, Corneal High-order Aberrations in 4mm diameter, Corneal Spherical aberration in 6 mm diameter , Uncorrected Visual Acuity, Best Corrected Visual Acuity. Ocular comorbidity, History of previous ocular surgery, B-scan ultrasound results, OCT scan results. Some information will also be provided by images, such as corneal topography, fundus photography，B-ultrasound and OCT, special attention needs to be paid to analyzing corneal regularity from these pictures. Based on the provided information, select the appropriate IOL type for the eye for surgery from the following options: ① Monofocal IOLs ② Bifocal IOLs ③ Extended depth of focus IOLs ④Trifocal IOLs ⑤ Monofocal toric IOLs ⑥ Bifocal toric IOLs ⑦ Trifocal toric IOLs
