# Supplementary material for: Assessing GPT-4o in cataract surgery decision-making: appropriateness, consistency, and clinical implications
Source: Front Artif Intell. 2026 May 29;9:1810899. doi: 10.3389/frai.2026.1810899 (PMC13260408; doi:10.3389/frai.2026.1810899)
Supplement: Supplementary file 3 [file Data_Sheet_3.docx]

**Supplementary Material 2. Clinical Decision Logic for IOL Reference Standard**

| 1. **Step 1: Ocular Surface & Cornea Assessment** |
| --- |
| If significant Ocular Surface Disease or irregular astigmatism (e.g., Keratoconus, post-refractive surgery) → **Monofocal IOL** is preferred; Multifocal/EDOF are contraindicated. |
| 1. **Step 2: Corneal Astigmatism (CYL)** |
| If Regular CYL 0.75 D → **Toric IOL** is recommended. |
| If Regular CYL < 0.75 D → **Non-Toric IOL**. |
| 1. **Step 3: Visual Demand & Lifestyle** |
| **High demand for spectacle independence (Near/Intermediate/Distance):** |
| If low angle alpha/kappa and low HOAs → **Trifocal or EDOF IOL**. |
| If the patient prioritizes night driving or has mild maculopathy → **EDOF or Monofocal Plus**. |
| **Standard visual demand:** |
| **Monofocal IOL** (target: emmetropia or monovision). |
| 1. **Step 4: Financial/Socioeconomic Factors** |
| Consideration of bifocal options in specific settings where trifocal/EDOF accessibility is limited, provided the patient accepts potential loss of intermediate vision. |
| 1. **Final Consensus**   The final "Reference Standard" is achieved when at least two senior consultants agree on the primary IOL category based on the above hierarchy. |
